# Changes in disease burden in Poland between 1990–2017 in comparison with other Central European countries: A systematic analysis for the Global Burden of Disease Study 2017

**DOI:** 10.1371/journal.pone.0226766

**Published:** 2020-03-02

**Authors:** Maria Gańczak, Tomasz Miazgowski, Marta Kożybska, Artur Kotwas, Marcin Korzeń, Bartosz Rudnicki, Tomasz Nogal, Catalina Liliana Andrei, Marcel Ausloos, Maciej Banach, Alexandra Brazinova, Maria-Magdalena Constantin, Eleonora Dubljanin, Claudiu Herteliu, Mihaela Hostiuc, Sorin Hostiuc, Mihajlo Jakovljevic, Jacek Jerzy Jozwiak, Katarzyna Kissimova-Skarbek, Zbigniew J. Król, Tomislav Mestrovic, Bartosz Miazgowski, Neda Milevska Kostova, Mohsen Naghavi, Ionut Negoi, Ruxandra Irina Negoi, Adrian Pana, Salvatore Rubino, Mario Sekerija, Radoslaw Sierpinski, Lucjan Szponar, Roman Topor-Madry, Isidora S. Vujcic, Justyna Widecka, Katarzyna Widecka, Bogdan Wojtyniak, Vesna Zadnik, Jacek A. Kopec

**Affiliations:** 1 Department of Infectious Diseases, Institute of Medical Sciences, Zielona Góra University, Zielona Góra, Poland; 2 Department of Propedeutics of Internal Diseases, Pomeranian Medical University, Szczecin, Poland; 3 Department of Medical Law of the Social Medicine Chair, Pomeranian Medical University in Szczecin, Faculty of Health Sciences, Szczecin, Poland; 4 Department of Public Health, Pomeranian Medical University, Faculty of Health Sciences, Szczecin, Poland; 5 Department of Methods of Artificial Intelligence and Applied Mathematics, West Pomeranian University of Technology, Szczecin, Poland; 6 Healthcare Management and Administration, Faculty of Health Sciences, Pomeranian Medical University, Szczecin, Poland; 7 Carol Davila University of Medicine and Pharmacy, Bucharest, Romania; 8 School of Business, University of Leicester, Leicester, England, United Kingdom; 9 Department of Statistics and Econometrics, Bucharest University of Economic Studies, Bucharest, Romania; 10 Department of Hypertension, Medical University of Lodz, Lodz, Poland; 11 Mothers’ Memorial Hospital Research Institute, Lodz, Poland; 12 Institute of Epidemiology, Faculty of Medicine, Comenius University, Bratislava, Slovakia; 13 IInd Department of Dermatology, Carol Davila University of Medicine and Pharmacy, Bucharest, Romania; 14 IInd Department of Dermatology, Colentina Clinical Hospital, Bucharest, Romania; 15 Faculty of Medicine, University of Belgrade, Belgrade, Serbia; 16 Department of General Surgery, Carol Davila University of Medicine and Pharmacy, Bucharest, Romania; 17 Department of Internal Medicine, Bucharest Emergency Hospital, Bucharest, Romania; 18 Department of Legal Medicine and Bioethics, Carol Davila University of Medicine and Pharmacy, Bucharest, Romania; 19 Department of Clinical Legal Medicine, National Institute of Legal Medicine Mina Minovici, Bucharest, Romania; 20 N.A. Semashko Department of Public Health and Healthcare, I.M. Sechenov First Moscow State Medical University (Sechenov University), Moscow, Russia; 21 Department of Family Medicine and Public Health, University of Opole, Opole, Poland; 22 Faculty of Medicine and Health Sciences, University of Opole, Opole, Poland; 23 Department of Health Economics and Social Security, Jagiellonian University Medical College, Krakow, Poland; 24 Data and Analyses Department, Ministry of Health, Warsaw, Poland; 25 Clinical Microbiology and Parasitology Unit, Dr. Zora Profozic Polyclinic, Zagreb, Croatia; 26 University Centre Varazdin, University North, Varazdin, Croatia; 27 Center for Innovation in Medical Education, Pomeranian Medical University, Szczecin, Poland; 28 Department of Health Policy and Management, Centre for Regional Policy Research and Cooperation ‘Studiorum’, Skopje, Macedonia; 29 Institute for Health Metrics and Evaluation, University of Washington, Seattle, WA, United States of America; 30 Department of Health Metrics Sciences, School of Medicine, University of Washington, Seattle, WA, United States of America; 31 General Surgery Department, Emergency Hospital of Bucharest, Bucharest, Romania; 32 Anatomy and Embryology Department, Carol Davila University of Medicine and Pharmacy, Bucharest, Romania; 33 Department of Cardiology, Cardio-Aid, Bucharest, Romania; 34 Department of Health Outcomes, Center for Health Outcomes & Evaluation, Bucharest, Romania; 35 Department of Biomedical Sciences, Università degli Studi di Sassari, Sassari, Italy; 36 Department of Medical Statistics, Epidemiology and Medical Informatics, School of Medicine, University of Zagreb, Zagreb, Croatia; 37 Croatian Institute of Public Health, Zagreb, Croatia; 38 Polish Medical Research Agency, Warsaw, Poland; 39 Department of Arrhythmias, Cardinal Wyszynski National Institute of Cardiology, Warsaw, Poland; 40 National Food and Nutrition Institute, Warsaw, Poland; 41 Institute of Public Health, Jagiellonian University Medical College, Krakow, Poland; 42 Agency for Health Technology Assessment and Tariff System, Warsaw, Poland; 43 Institute of Epidemiology, University of Belgrade, Belgrade, Serbia; 44 Zdroje Hospital, Pomeranian Medical University, Szczecin, Poland; 45 Cardiology Clinic, Pomeranian Medical University, Szczecin, Poland; 46 Department of Population Health Monitoring and Analysis, National Institute of Public Health, Warsaw, Poland; 47 Epidemiology and Cancer Registry Sector, Institute of Oncology Ljubljana, Ljubljana, Slovenia; 48 University of British Columbia, Vancouver, BC, Canada; 49 Arthritis Research Canada, Richmond, BC, Canada; University of Central Florida, UNITED STATES

## Abstract

**Background:**

Systematic collection of mortality/morbidity data over time is crucial for monitoring trends in population health, developing health policies, assessing the impact of health programs. In Poland, a comprehensive analysis describing trends in disease burden for major conditions has never been published. The Global Burden of Diseases, Injuries, and Risk Factors Study (GBD) provides data on the burden of over 300 diseases in 195 countries since 1990. We used the GBD database to undertake an assessment of disease burden in Poland, evaluate changes in population health between 1990–2017, and compare Poland with other Central European (CE) countries.

**Methods:**

The results of GBD 2017 for 1990 and 2017 for Poland and CE were used to assess rates and trends in years of life lost (YLLs), years lived with disability (YLDs), disability-adjusted life years (DALYs). Data came from cause-of-death registration systems, population health surveys, disease registries, hospitalization databases, and the scientific literature. Analytical approaches have been used to adjust for missing data, errors in cause-of-death certification, and differences in data collection methodology. Main estimation strategies were ensemble modelling for mortality and Bayesian meta-regression for disability.

**Results:**

Between 1990–2017, age-standardized YLL rates for all causes declined in Poland by 46.0% (95% UI: 43.7–48.2), YLD rates declined by 4.0% (4.2–4.9), DALY rates by 31.7% (29.2–34.4). For both YLLs and YLDs, greater relative declines were observed for females. There was a large decrease in communicable, maternal, neonatal, and nutritional disease DALYs (48.2%; 46.3–50.4). DALYs due to non-communicable diseases (NCDs) decreased slightly (2.0%; 0.1–4.6). In 2017, Poland performed better than CE as a whole (ranked fourth for YLLs, sixth for YLDs, and fifth for DALYs) and achieved greater reductions in YLLs and DALYs than most CE countries. In 2017 and 1990, the leading cause of YLLs and DALYs in Poland and CE was ischaemic heart disease (IHD), and the leading cause of YLDs was low back pain. In 2017, the top 20 causes of YLLs and YLDs in Poland and CE were the same, although in different order. In Poland, age-standardized DALYs from neonatal causes, other cardiovascular and circulatory diseases, and road injuries declined substantially between 1990–2017, while alcohol use disorders and chronic liver diseases increased. The highest observed-to-expected ratios were seen for alcohol use disorders for YLLs, neonatal sepsis for YLDs, and falls for DALYs (3.21, 2.65, and 2.03, respectively).

**Conclusions:**

There was relatively little geographical variation in premature death and disability in CE in 2017, although some between-country differences existed. Health in Poland has been improving since 1990; in 2017 Poland outperformed CE as a whole for YLLs, YLDs, and DALYs. While the health gap between Poland and Western Europe has diminished, it remains substantial. The shift to NCDs and chronic disability, together with marked between-gender health inequalities, poses a challenge for the Polish health-care system. IHD is still the leading cause of disease burden in Poland, but DALYs from IHD are declining. To further reduce disease burden, an integrated response focused on NCDs and population groups with disproportionally high burden is needed.

## Introduction

Central Europe (CE) is a geographical region composed mostly of ex-communist countries and including Albania, Bosnia and Herzegovina, Bulgaria, Croatia, Czech Republic, Hungary, North Macedonia, Montenegro, Poland, Romania, Serbia, Slovakia, and Slovenia. Since the 1990s, despite many earlier similarities in economic development and the organization of health care systems, this group of countries have been facing numerous health challenges as a result of the shift from previous centrally planned economies to market economies, the formation of new countries within the same territories, local conflicts, aging, as well as the adoption of some unhealthy elements of Western lifestyle [1]. Since 2004, the majority of CE countries have become members of the European Union, with the exception of Albania, North Macedonia, Montenegro, and Serbia (candidate countries), and Bosnia and Herzegovina (potential candidate). Although the region has historically seen overall improvements in life expectancy and other health indicators, there are still high disparities in their gross domestic product, socio-demographic profiles, public health expenditures, health care coverage, and Human Development Index (HDI) [2]. The HDI, which is a summary measure (between 0 and 1) for assessing progress in three basic dimensions of human development, i.e., a long and healthy life, access to knowledge, and a decent standard of living, varied markedly in 2018 across CE countries, ranging from 0.768 to 0.896, while its mean value in Western Europe reached 0.920 [2].

In Poland, the biggest country in this region with a population of over 38 million, mortality due to cardiovascular disease, cancer, and injuries–the three leading causes of premature death–are gradually in decline, while life expectancy at birth is increasing [3, 4]. However, despite a high HDI (0.865 in 2017), total health expenditure in Poland, expressed as a percentage of gross domestic product (GDP), is one of the lowest in CE (6.5% in 2016) and much lower than in the European Union as a whole (8.4%) [5]. As the Polish population grows older, similar to other European populations [3, 4, 6], the number of years Poles can expect to live with disability and impaired quality of life from chronic conditions has increased [6, 7]. This suggests that declines in mortality have not been matched by similar declines in morbidity, resulting in people living longer with diseases. Indeed, a recent national report in Poland [7] showed an increasing prevalence of lower back pain, high blood pressure, neck pain, and osteoarthritis, suggesting that non-fatal outcomes of diseases along with injuries are becoming a larger component of the total burden of disease.

Previous publications on the health status of Poles [3, 6, 8], based on routinely collected mortality and morbidity statistics and national surveys, are limited in scope and timeframe, and do not allow for a comprehensive comparison with other countries. In this study, we carried out a systematic analysis of data from the Global Burden of Diseases, Injuries, and Risk Factors (GBD) Study [9–12], which provides the most comprehensive picture of health loss across countries to date. The purpose of the study was to present recent estimates of disease burden for a range of non-communicable and communicable diseases and injures, track trends and benchmark progress in disease burden reduction between 1990 and 2017, and compare observed measures of disease burden to those expected on the basis the Socio-demographic Index (SDI), a summary measure of a geography’s socio-demographic development [13]. By considering the varying levels of development across the CE countries, the analyses reported here make it possible to evaluate each country’s performance relative to its peers. Hence, this report extends the results of previous research by using an improved methodological approach to better understand the burden of disease at a regional level and provides data that are relevant to policy-makers.

## Methods

The GBD is a large-scale scientific collaboration whose goal is to generate valid, comparable, and up- to-date information on mortality, morbidity, and risk factors for all countries over time [14]. The results are regularly updated by new data to improve the accuracy of past estimates. The GBD database provides access to a complete set of age, national and subnational, and gender-specific estimates of burden across a wide range of causes. In the 2017 iteration of the GBD (GBD 2017), disease burden was estimated for 359 diseases (282 causes of death) from 1990 until 2017 for 195 countries [9–12, 15]. Disease burden in GBD is measured with several population health indicators, including standard measures of disease frequency, such as death rates, disease incidence and prevalence, and life expectancy, and more complex indicators, such as years of life lost (YLLs), years lived with disability (YLDs), and disability-adjusted life years (DALYs). YLLs are a measure of premature mortality. They are defined as the number of deaths from a disease multiplied by standard expected years of life at the time of death [9]. Standard expected years of life are obtained for each age from the country (population >5 million) with the highest life expectancy for that age, male or female [9]. YLDs are a measure of years of life lived with disability, defined as the number of people with a given disease (prevalence) multiplied by the corresponding disability weight, with different weights used for different disease stages or sequelae [11]. Finally, DALYs are defined as a sum of YLLs and YLDs and are considered an overall measure of population health that combines mortality and morbidity [12].

For the current study, we extracted GBD data for the period 1990 to 2017 for Poland, other CE countries, and the CE, Eastern Europe (Belarus, Estonia, Latvia, Lithuania, Moldova, Russian Federation, Ukraine), and Western Europe (Andorra, Austria, Belgium, Cyprus, Denmark, Finland, France, Germany, Greece, Iceland, Ireland, Israel, Italy, Luxembourg, Malta, Netherlands, Norway, Portugal, Switzerland, Spain, Sweden, United Kingdom) regions. Data on levels and trends in health had been provided by various data systems, including health surveys [16], national registration of causes of death [17], infectious disease surveillance [18, 19], cancer registries [20], hospitalization data [21], and demographic censuses [16]. Additional data were obtained from the scientific literature, other published reports, and other sources [9–12, 15]. The data were systematically collected, reviewed, and adjusted for missing data, errors in cause of death certification, and differences in disease definitions and data collection methodology across different data sources and locations, to produce valid, reproducible, and comparable estimates of disease burden. Death rates by age, gender, year, and location for most diseases were estimated using a statistical procedure developed for the GBD, the Cause of Death Ensemble model (CODEm) [10]. In ensemble modeling, the best prediction model for death rates is found as a weighted combination of a range of different models. These models are developed by systematically testing a large number of functional forms and combinations of covariates. The best performing models are selected based on out-of-sample predictive ability. Non-fatal outcomes were modeled with DisMod-MR 2.1, a Bayesian meta-regression tool developed specifically for the GBD. DisMod-MR 2.1 uses a compartmental model that combines information from different sources and generates estimates of epidemiological parameters adjusted for gaps and inconsistencies in the data. The parameters are estimated using Markov Chain Monte Carlo and Bayesian statistical methods [11, 22]. Details of data sources, bias-reduction methods, and statistical analyses are described in GBD capstone papers [9–12, 15] and other publications [22, 23].

In this study, we describe disease burden in terms of YLLs, YLDs, and DALYs. To assess the impact of aging on disease burden, we present both all-age (non-standardized) rates and age-standardized rates based on the GBD standard world population. We display the trends over time, describe the rates according to age and gender, and provide comparative data for CE as a whole as well as Western Europe (WE) and Eastern Europe (EE). In the GBD, diseases are grouped into hierarchically organized categories according to a 4-level classification system. Level 1 has only three broad categories (non-communicable diseases, communicable, maternal, neonatal, and nutritional diseases, and injuries). There are 22 disease groups at Level 2 (e.g., cardiovascular diseases), 169 conditions at Level 3 (e.g., stroke) and 293 at Level 4 (e.g., intracerebral hemorrhage) [9–10]. With a few exceptions, we show disease-specific rates for Level 3 of the GBD hierarchy. Since conventional confidence intervals are not available for GBD estimates, 95% uncertainty intervals (UI) were generated by taking 1,000 draws from the posterior distribution of each estimate, with upper and lower bounds determined by the 25th and 975th values of the draws [9, 23].

The GBD Study groups countries according to their SDI. The SDI is defined as a combination (geometric mean) of lag distributed income per capita, average years of education for persons 15 years of age or older, and fertility rate for women less than 25 years of age [11]. For each country and disease burden indicator, the expected value for a given year is calculated based on that country’s SDI and a model of the relationship between SDI and disease burden. We used the observed-to-expected ratios (OER) to assess how Poland is performing compared to its peers, i.e., other countries at a similar level of development.

The study utilized existing data and, therefore, does not require ethical approval. The GBD study complies with the Guidelines for Accurate and Transparent Health Estimates Reporting (GATHER) recommendations [24].

## Results

### Years of life lost (YLLs)

#### Overview

**Fig 1:**
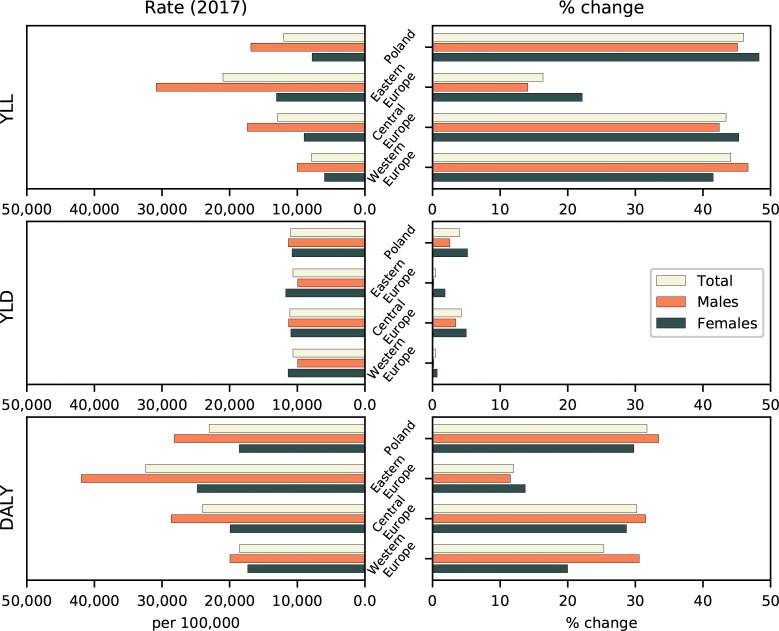
Age-standardized rates in 2017 (left) and relative (%) change from 1990 to 2017 (right) for YLLs, YLDs, and DALYs for males, females, and both sexes combined for Poland, Eastern Europe, Central Europe, and Western Europe.

Estimates of age-standardized YLL rates and ranking for all CE countries for the years 1990 and 2017 are shown in Table 1, and percentage changes for Poland, CE, EE, and WE are shown in Fig 1. Corresponding estimates of all-age YLLs are given in the S1 Table. Between 1990 and 2017, the age-standardized YLL rate in Poland decreased by 46.0%, from 22,254 (95% UI: 22,166–22,346) to 12,007 (11,475–12,585) per 100,000. The rate decreased by 45.1% in males and 48.3% in females (Fig 1). For comparison, the YLL rate in CE as a whole decreased by 43.4%, from 22,814 (22,752–22,879) to 12,910 (12,665–13,151), 42.4% in males and 45.3% in females. Of all CE countries, only Slovenia (52.9% in both males and females), the Czech Republic (54.4% and 50.9%, respectively), and Hungary (48.1% and 48.3%, respectively) experienced greater declines in age-standardized YLL rates in the above mentioned period. Other CE countries also experienced a reduction in YLL rates, although not as large. The smallest decrease was observed in Montenegro (24.4%). In 2017, Poland performed better than CE as a whole and achieved a greater improvement in rank since 1990 (from ninth to fourth) than any other CE country but Slovakia (from 11th to sixth).

**Table 1:** Age-standardized YLL, YLD, and DALY rates (95% UI) and country ranks (from best to worst) for Poland and other Central European countries, for both sexes combined, males, and females, in 1990 and 2017.

Country	Age-standardized YLL rate per 100,000				Age-standardized YLD rate per 100,000				Age-standardized DALY rate per 100,000			
	1990		2017		1990		2017		1990		2017	
	Rate 95% UI	Rank	Rate 95% UI	Rank	Rate 95% UI	Rank	Rate 95% UI	Rank	Rate 95% UI	Rank	Rate 95% UI	Rank
**Total**												
**Central Europe**	**22,814**		**12,910**		**11,593**		**11,099**		**34,407**		**24,009**	
	**22,752–22,879**		**12,665–13,151**		**8,678–14,879**		**8,304–14,269**		**31,476–37,687**		**21,159–27,224**	
Albania	20,416	5	12,358	5	11,081	3	10,656	1	31,497	5	23,014	4
	19,655–21,197		10,677–14,253		8,270–14,301		7,965–13,666		28,522–34,764		19,681–26,573	
Bosnia	17,971	3	12,934	7	11,489	10	11,368	13	29,460	3	24,302	10
	17,750–18,198		12,300–13,566		8,600–14,739		8,547–14,526		26,592–32,720		21,374–27,583	
Bulgaria	21,413	7	15,799	13	11,150	5	10,743	2	32,562	6	26,542	12
	21,240–21,601		14,998–16,607		8,268–14,357		8,015–13,881		29,717–35,723		23,670–29,676	
Croatia	19,455	4	10,763	3	11,065	2	10,836	3	30,519	4	21,599	3
	19,252–19,669		10,239–11,310		8,270–14,187		8,129–13,928		27,782–33,689		18,978–24,735	
Czech Republic	21,232	6	10,093	2	11,465	7	11,289	11	32,697	7	21,382	2
	21,075–21,391		9,609–10,616		8,543–14,829		8,457–14,631		29,851–35,985		18,556–24,811	
Hungary	25,258	10	13,008	8	12,014	12	11,244	9	37,272	12	24,252	9
	25,079–25,434		12,431–13,645		9,059–15,415		8,481–14,435		34,318–40,661		21,338–27,319	
N. Macedonia	22,171	8	13,081	10	11,147	4	10,909	4	33,319	8	23,990	8
	21,773–22,559		12,475–13,712		8,344–14,326		8,178–14,048		30,522–36,528		21,211–27,176	
Montenegro	17,255	1	13,042	9	10,895	1	10,934	5	28,150	1	23,976	7
	16,762–17,768		12,176–13,971		8,172–14,095		8,166–14,011		25,356–31,326		21,174–27,265	
**Poland**	**22,254**	**9**	**12,007**	**4**	**11,487**	**9**	**11,033**	**6**	**33,741.12**	**9**	**23,040**	**5**
	**22,166–22,346**		**11,475–12,585**		**8,581–14,864**		**8,222–14,129**		**30,817–37,080**		**20,212–26,249**	
Romania	25,274	13	15,553	12	12,090	13	11,255	10	37,363	13	26,808	13
	25,141–25,421		14,865–16,229		9,066–15,492		8,489–14,525		34,279–40,771		23,876–30,259	
Serbia	23,746	12	14,382	11	11,330	6	11,086	7	35,076	11	25,468	11
	23,514–23,967		13,733–15,007		8,495–14,595		8,319–14,247		32,212–38,318		22,684–28,807	
Slovakia	22,272	11	12,376	6	11,470	8	11,091	8	33,741.48	10	23,466	6
	22,077–22,479		11,765–13,031		8,578–14,793		8,314–14,282		30,840–37,072		20,562–26,795	
Slovenia	17,472	2	8,413	1	11,560	11	11,290	12	29,032	2	19,703	1
	17,232–17,702		7,909–8,954		8,623–14,829		8,443–14,528		26,074–32,329		16,907–23,034	
**Males**												
**Central Europe**	**30,187**		**17,385**		**11,678**		**11,278**		**41,864**		**28,662**	
	**30,098–30,272**		**16,933–17,814**		**8,767–14,985**		**8,489–14,446**		**38,905–45,161**		**25,786–31,912**	
Albania	25,499	4	16,473	6	11,077	2	10,730	1	36,576	4	27,204	4
	24,643–26,395		13,501–19,697		8,251–14,222		8,009–13,783		33,654–39,816		22,963–31,677	
Bosnia	22,966	2	16,217	4	11,415	7	11,620	13	34,381	2	27,837	6
	22,659–23,267		15,216–17,296		8,562–14,565		8,755–14,738		31,566–37,611		24,816–31,218	
Bulgaria	28,082	7	21,177	13	11,289	6	10,826	2	39,371	7	32,004	12
	27,841–28,342		19,842–22,524		8,412–14,496		8,105–13,974		36,427–42,567		29,054–35,361	
Croatia	26,440	6	14,587	3	11,225	4	11,073	5	37,664	6	25,660	3
	26,167–26,730		13,649–15,539		8,398–14,371		8,296–14,185		34,822–40,903		22,906–28,866	
Czech Republic	29,148	8	13,297	2	11,468	8	11,497	11	40,616	8	24,794	2
	28,938–29,371		12,422–14,211		8,601–14,810		8,640–14,811		37,752–43,974		21,801–28,233	
Hungary	34,231	13	17,763	11	12,013	12	11,335	9	46,244	13	29,098	11
	33,984–34,481		16,726–18,952		9,075–15,403		8,585–14,551		43,284–49,640		26,111–32,381	
N. Macedonia	25,974	5	17,103	9	11,211	3	11,067	4	37,185	5	28,169	8
	25,493–26,454		16,021–18,193		8,354–14,380		8,280–11,201		34,407–40,437		25,174–31,583	
Montenegro	22,155	1	16,334	5	10,961	1	11,005	3	33,116	1	27,339	5
	21,514–22,816		14,783–18,030		8,217–14,127		8,209–14,045		30,239–36,267		24,205–30,925	
**Poland**	**30,723**	**9**	**16,859**	**8**	**11,629**	**9**	**11,329**	**8**	**42,352**	**10**	**28,189**	**9**
	**30,604–30,849**		**15,886–17,913**		**8,700–14,952**		**8,502–14,568**		**39,440–45,708**		**25,080–31,592**	
Romania	31,464	12	20,923	12	12,181	13	11,299	7	43,645	12	32,222	13
	31,275–31,665		19,747–22,222		9,163–15,614		8,468–14,597		40,598–47,082		29,074–35,637	
Serbia	30,869	11	17,172	10	11,283	5	11,114	6	42,153	9	28,286	10
	30,570–31,183		16,116–18,198		8,457–14,443		8,342–14,243		39,334–45,345		25,373–31,735	
Slovakia	30,729	10	16,593	7	11,755	10	11,375	10	42,484	11	27,968	7
	30,448–31,028		15,590–17,597		8,819–15,133		8,527–14,666		39,443–45,914		24,961–31,496	
Slovenia	24,385	3	11,497	1	11,803	11	11,522	12	36,188	3	23,019	1
	24,047–24,760		10,635–12,328		8,794–15,144		8,617–14,787		33,250–39,646		20,091–26,322	
**Females**												
**Central Europe**	**16,427**		**8,986**		**11,528**		**10,952**		**27,954**		**19,938**	
	**16,361–16,499**		**8,746–9,229**		**8,582–14,898**		**8,177–14,163**		**25,020–31,298**		**17,134–23,081**	
Albania	15,944	9	8,580	5	11,103	6	10,597	1	27,047	9	19,177	5
	15,256–16,653		6,939–10,395		8,273–14,469		7,929–13,687		24,082–30,284		15,963–22,625	
Bosnia	13,673	3	9,993	9	11,589	12	11,143	11	25,262	4	21,136	10
	13,445–13,912		9,306–10,738		8,699–14,844		8,354–14,328		22,352–28,585		18,265–24,307	
Bulgaria	15,343	8	11,057	12	11,031	4	10,689	3	26,373	7	21,746	11
	15,160–15,544		10,324–11,847		8,210–14,273		7,992–13,820		23,560–29,565		18,855–24,784	
Croatia	13,748	4	7,458	3	10,947	2	10,626	2	24,695	3	18,084	2
	13,508–13,979		6,926–8,027		8,191–14,039		7,980–13,665		21,915–27,839		15,398–21,153	
Czech Republic	14,783	5	7,255	2	11,472	11	11,115	10	26,254	5	18,369	3
	14,622–14,955		6,765–7,797		8,580–14,845		8,283–14,431		23,422–29,535		15,503–21,699	
Hungary	17,714	11	9,156	7	12,022	3	11,193	12	29,736	12	20,349	7
	17,528–17,894		8,549–9,844		8,981–15,513		8,390–14,452		26,741–33,197		17,472–23,466	
N. Macedonia	18,623	12	9,687	8	11,090	5	10,771.53	4	29,713	11	20,459	8
	18,180–19,056		9,092–10,293		8,266–14,279		8,048–13,921		26,850–32,886		17,629–23,663	
Montenegro	12,961	2	10,080	10	10,866	1	10,878	7	23,816	2	20,958	9
	12,492–13,463		9,297–10,940		8,110–14,103		8,165–14,079		21,032–27,006		18,005–24,110	
**Poland**	**15,102**	**7**	**7,810**	**4**	**11,361**	**8**	**10,772.41**	**5**	**26,464**	**8**	**18,582**	**4**
	**15,005–15,210**		**7,315–8,340**		**8,432–14,753**		**8,051–13,865**		**23,567–29,802**		**15,853–21,677**	
Romania	19,541	13	10,758	11	12,023	13	11,251	13	31,564	13	22,009	12
	19,366–19,718		10,100–11,408		8,994–15,538		8,443–14,586		28,493–35,044		19,169–25,402	
Serbia	17,584	10	11,612	13	11,400	10	11,067	8	28,983	10	22,680	13
	17,363–17,802		10,815–12,365		8,569–14,703		8,344–14,245		26,160–32,301		19,928–25,785	
Slovakia	15,066	6	8,811	6	11,234	7	10,858	6	26,300	6	19,669	6
	14,841–15,280		8,180–9,478		8,386–14,568		8,128–14,050		23,439–29,659		17,003–22,833	
Slovenia	11,971	1	5,643	1	11,364	9	11,073	9	23,336	1	16,716	1
	11,686–12,264		5,144–6,179		8,491–14,658		8,247–14,277		20,436–26,591		13,905–19,992	

In 2017, age-standardized YLL rates in WE were much lower than in Poland, at 7,901 per 100,000 overall (95% UI: 7,728–8,082), 10,014 (9,717–10,336) in males and 5,985 (5,792–6,190) in females (Fig 1). In EE, on the other hand, the rates were higher, at 20,995 (20,752–21,243) overall, 30,859 (30,391–31,306) in males and 13,076 (12,870–13,290) in females. YLL rates in WE declined by 44.1% (46.7% in males and 41.5% in females) between 1990 and 2017. In the same period, the rates in EE declined by only 16.4% (14.1% and 22.1%, respectively).

#### Main causes of YLLs

Looking at Level 1 causes, communicable, maternal, neonatal, and nutritional diseases (CMNN) contributed 4.2% (95% UI: 3.9–4.4) to total all-age YLLs in Poland in 2017, non-communicable diseases (NCD) contributed 85.7% (85.2–86.1), and injuries 10.2% (9.9–10.5) (Table 2). Significant progress has been made for CMNN, which declined by 61.6% between 1990 and 2017, from 1,996.5 to 767.2 per 100,000, as well as for injuries (38.9% decline, from 3,078.4 to 1,881.1), and NCDs (13.8% decline, from 18,348.2 to 15,825.5). In CE, CMNN causes contributed 4.1% (4.0–4.2) to total YLLs in 2017, NCDs contributed 87.6% (87.5–87.8), and injuries 8.3% (8.1–8.4). All-age YLL rates for CMNN causes fell by 65.3%, from 2,406.5 per 100,000 in 1990 to 834.9 in 2017, whereas those from injuries fell by 45.2%, from 3,061.1 to 1,677.8, and those from NCDs by 6.5%, from 19,068.0 to 17,821.8. YLL rates for 22 major groups of conditions (Level 2 of the GBD hierarchy) are provided in the S2 Table.

**Table 2:** All-age rates, percentage contribution, and relative (%) change for Level 1 causes of YLLs, YLDs, and DALYs for Poland and Central Europe, both sexes combined, in 1990 and 2017.

Cause	All-age YLL rate				All-age YLD rate				All-age DALY rate			
	1990		2017		1990		2017		1990		2017	
	Rate per 100,000	Contribution % (95% UI)	Rate per 100,000	Change %	Rate per 100,000	Contribution % (95% UI)	Rate per 100,000	Change %	Rate per 100,000	Contribution % (95% UI)	Rate per 100,000	Change %
Poland												
CMMN	1,996.5	4.2 3.9–4.4	767.2	-61.6	1,048.4	5.7 4.5–7.1	810.2	-22.7	3,044.8	4.8 4.3–5.4	1,577.4	-48.2
NCDs	18,348.2	85.7 85.2–86.1	15,825.5	-13.8	9,179.5	78.2 75.9–80.2	11,154.3	21.5	27,527.7	82.4 81.3–83.5	26,980.0	-2.0
Injuries	3,078.4	10.2 9.9–10.5	1,881.1	-38.9	2,010.2	16.1 14.4–18.1	2,303.1	14.6	5,088.6	12.7 11.8–13.9	4,184.2	-17.8
Central Europe												
CMMN	2,406.5	4.1 4.0–4.2	834.9	-65.31	1,055.4	5.8 4.9–7.0	839.3	-20.5	3,461.8	4.8 4.4–5.2	1,674.1	-51.6
NCDs	19,068.0	87.6 87.5–87.8	17,821.8	-6.5	9,508.9	78.5 76.3–80.6	11,364.0	19.5	28,576.8	83.9 82.7–84.9	29,185.8	2.1
Injuries	3,061.1	8.3 8.1–8.4	1,677.8	-45.2	2,047.2	15.7 14.0–17.6	2,270.7	10.9	5,108.3	11.3 10.4–12.4	3,948.4	-22.7

Table 3 lists the top 25 Level 3 causes of age-standardized YLLs for Poland in 2017, compared to 1990, for both sexes combined, as well as percentage changes in counts, all-age rates, and age-standardized rates. In 2017, the top five causes were ischemic heart disease (IHD), lung cancer, stroke, self-harm, and colorectal cancer. Age-standardized rates decreased substantially between 1990 and 2017 for IHD (63.3%) and stroke (51.2%), somewhat less for lung cancer (15.4%) and only slightly for colorectal cancer (6.7%); however, rates remained virtually unchanged for self-harm. The remaining top 10 causes of YLLs were road injuries, chronic liver diseases, neonatal disorders, lower respiratory infections, and Alzheimer’s disease. Of those, age-standardized rates increased (33.9%) for chronic liver diseases.

**Table 3:** Top 25 Level 3 causes of YLLs, YLDs, and DALYs in Poland in 2017 for both sexes combined, males, and females, and changes in ranks, counts, all-age rates, and age-standardized rates between 1990 and 2017.

**TOTAL**															
**#**	**YLLs**	**change in rank**	**% change in counts**	**%** **change in all-age** **rates**	**% change in age-standar-dized rates**	**YLDs**	**change in rank**	**%** **change in counts**	**%** **change in all-age** **rates**	**% change in age-standar-dized rates**	**DALYs**	**change in rank**	**%** **change in counts**	**%** **change in all-age** **rates**	**% change in age-standar-dized rates**
1.	Ischemic heart disease	0	-42.5	-41.5	-63.3	Low back pain	0	25.6	27.8	1.3	Ischemic heart disease	0	-41.0	-40.0	-62.2
2.	Lung cancer	+3	24.7	26.9	-15.4	Falls	0	16.6	18.6	-7.6	Low back pain	+4	25.6	27.8	1.3
3.	Stroke	-1	-25.1	-23.8	-51.2	Headache disorders	0	5.7	7.6	0.02	Falls	+1	10.8	12.8	-13.0
4.	Self-harm	+3	6.9	8.7	0.3	Neonatal disorders	0	-4.0	-2.3	6.5	Stroke	-1	-17.0	-15.6	-45.8
5.	Colorectal cancer	+4	38.3	40.7	-6.7	Diabetes	+2	62.9	65.8	21.2	Neonatal disorders	-3	-59.2	-58.5	-56.9
6.	Road injuries	-2	-62.2	-61.5	-62.2	Age-related hearing loss	-1	35.2	37.5	-2.9	Lung cancer	+2	24.8	27.0	-15.3
7.	Chronic liver diseases	+8	69.6	72.6	33.9	Depressive disorders	+1	13.7	15.7	-0.9	Headache disorders	+3	5.7	7.6	0.02
8.	Neonatal disorders	-5	-85.8	-84.8	-77.3	Anxiety disorders	+3	4.4	6.2	-0.01	Road injuries	-3	-48.0	-47.0	-52.5
9.	Lower respiratory infect.	-1	-9.9	-8.3	-42.4	Neck pain	+3	25.9	28.1	0.05	Diabetes	+2	27.9	32.0	-5.0
10.	Alzheimer’s disease	+2	65.1	67.9	-13.1	COPD	-4	10.2	12.1	-24.5	Self-harm	+2	7.0	8.8	0.22
11.	Alcohol use disorders	+9	51.5	54.1	27.9	Blindness and vision impairment	-1	21.6	23.7	-10.1	COPD	-2	-3.7	-2.0	-35.7
12.	Congenital defects	-6	-78.8	-78.4	-69.9	Oral disorders	-3	11.4	13.3	-12.8	Alcohol use disorders	+5	39.0	41.4	20.2
13.	Cardiomyopathy	+1	33.6	35.9	-11.8	Upper digestive diseases	+2	4.3	6.1	-6.0	Congenital defects	-6	-61.8	-61.1	-58.2
14.	COPD	-4	-15.7	-14.3	-45.6	Stroke	-1	31.3	33.6	-13.2	Chronic liver diseases	+9	72.0	75.0	36.2
15.	Breast cancer	+3	12.3	14.3	-19.4	Road injuries	-1	2.3	4.1	-14.8	Colorectal cancer	+1	39.6 67.8	42.0	-5.8
16.	Stomach cancer	-5	-30.5	-29.3	-51.5	Congenital defects	0	-3.9	-2.3	-1.5	Alzheimer’s disease	-2		70.7	-17.7
17.	Pancreatic cancer	+6	29.9	32.1	-10.6	Alcohol use disorders	-3	20.5	22.6	9.3	Age-related hearing loss	+1	35.2	37.5	-2.9
18.	Falls	-1	-12.3	-10.7	34.6	Mechanical forces	0	14.4	16.4	-3.4	Depressive disorders	+4	13.7	15.7	-0.9
19.	Diabetes	-3	-12.3	-10.7	-39.2	Asthma	-2	-9.7	-8.2	-11.4	Lower respiratory infections	-6	-9.9	-8.4	-42.1
20.	Brain cancer	+5	8.2	10.1	-12.7	Dermatitis	+2	-11.4	-9.9	1.0	Upper digestive diseases	0	2.7	4.4	-12.7
21.	Leukemia	+5	-5.3	-3.7	-29.5	Gynecological diseases	0	-3.5	-1.9	-5.9	Cardiomyopathy	0	36.1	38.5	-10.1
22.	Other malignant neoplasms	+2	-19.4	-18.0	-37.9	Other mental disorders	+2	14.6	16.6	0.5	Anxiety disorders	+5	4.4	6.2	-0.01
23.	Ovarian cancer	+10	26.7	28.9	-9.6	Drug use disorders	+4	11.8	13.8	11.0	Neck pain	+6	25.9	28.1	0.05
24.	Prostate cancer	+16	85.2	88.4	18.7	Bipolar disorder	+1	5.1	6.9	-0.04	Blindness and vision impairment	+1	21.6	23.7	-10.1
25.	Kidney cancer	+14	52.5	55.2	5.9	Schizophrenia	+3	17.1	19.1	3.9	Oral disorders	-1	11.4	13.3	-12.8
**MALES**															
**#**	**YLLs**	**change in rank**	**%** **change in counts**	**%** **change in all-age** **rates**	**% change in age-standar-dized rates**	**YLDs**	**change in rank**	**%** **change in counts**	**%** **change in all-age** **rates**	**% change in age-standar-dized rates**	**DALYs**	**change in rank**	**%** **change in counts**	**%** **change in all-age** **rates**	**% change in age-standar-dized rates**
1.	Ischemic heart disease	0	-44.3	-42.9	-63.4	Falls	0	16.5	19.4	-5.3	Ischemic heart disease	0	-43.1	-41.6	-62.6
2.	Lung cancer	0	5.3	8.0	-29.6	Low back pain	0	26.7	29.9	2.6	Falls	+4	10.8	13.6	-10.8
3.	Self-harm	+3	11.7	14.5	3.9	Neonatal disorders	+1	-2.0	0.5	10.7	Lung cancer	+2	5.3	8.0	-29.5
4.	Stroke	-1	-17.4	-15.3	-45.0	Headache disorders	-1	5.2	7.9	0.1	Stroke	-1	-10.5	-8.3	-40.3
5.	Road injuries	0	-63.1	-62.2	-63.7	Diabetes	+1	74.8	79.2	28.6	Low back pain	+3	26.6	29.8	2.5
6.	Chronic liver diseases	+8	78.6	83.2	34.8	Age-related hearing loss	-1	32.1	35.4	-3.8	Self-harm	+4	11.7	14.5	3.8
7.	Alcohol use disorders	+6	45.7	49.4	22.0	Depressive disorders	+3	17.1	20.1	5.3	Neonatal disorders	-5	-61.8	-60.8	-58.6
8.	Colorectal cancer	+4	59.7	63.7	6.2	Road injuries	0	-3.9	-1.4	-20.0	Road injuries	-4	-52.7	-51.5	-56.2
9.	Lower respiratory infections	0	-7.1	-4.7	-36.5	Blindness and vision impairment	0	19.9	23.0	-10.1	Alcohol use disorders	+3	37.2	40.6	17.2
10.	Cardiomyopathy	+7	45.8	49.5	-1.8	COPD	-3	8.5	11.3	-26.7	Diabetes	+3	50.3	54.1	9.2
11.	Neonatal disorders	-7	-85.4	-85.1	-77.8	Mechanical forces	+1	15.6	18.5	-3.5	Chronic liver diseases	+7	81.4	86.0	37.4
12.	COPD	-4	-26.8	-24.9	-53.1	Alcohol use disorders	+3	21.4	24.5	8.6	COPD	-3	-15.2	-13.0	-44.6
13.	Congenital defects	-6	-78.3	-77.7	-69.5	Neck pain	+1	26.6	29.8	0.0	Colorectal cancer	+4	61.2	65.3	7.1
14.	Alzheimer's disease	+4	67.2	71.5	-12.1	Stroke	-1	38.2	41.7	-7.2	Headache disorders	+2	5.2	7.8	0.1
15.	Falls	+1	-7.1	-4.8	-28.0	Oral disorders	-4	13.8	16.7	-11.1	Congenital defects	-8	-62.9	-62.0	-58.3
16.	Stomach cancer	-6	-28.1	-26.2	-51.5	Upper digestive diseases	0	10.7	13.5	-1.2	Lower respiratory infections	-5	-7.1	-4.7	-36.2
17.	Prostate cancer	-6	85.2	89.9	14.3	Anxiety disorders	0	2.3	4.9	0.3	Cardiomyopathy	+3	47.4	51.1	-0.6
18.	Diabetes	+2	15.0	17.9	-19.0	Congenital defects	0	-5.7	-3.3	-1.6	Age-related hearing loss	+5	32.0	35.4	-3.8
19.	Pancreatic cancer	+3	27.0	30.3	-13.8	Drug use disorders	+1	12.4	15.2	11.6	Alzheimer’s disease	0	69.4	73.7	-10.9
20.	Brain cancer	+9	10.3	13.1	-10.4	Asthma	-1	-15.8	-13.7	-14.7	Upper digestive diseases	+4	3.5	6.1	-14.3
21.	Bladder cancer	+10	54.0	57.9	-0.3	Other mental disorders	0	14.1	17.0	0.0	Mechanical forces	0	-10.9	-8.7	-25.1
22.	Leukemia	+3	-0.6	2.0	-24.9	Dermatitis	+1	-11.9	-9.6	1.1	Depressive disorders	+6	17.1	20.0	5.3
23.	Drowning	-8	-62.0	-70.0	-63.9	Bipolar disorder	+2	5.2	7.9	-0.1	Stomach cancer	-8	-27.9	-26.1	-51.4
24.	Kidney cancer	+15	62.0	66.1	12.4	Ischemic heart disease	-2	17.2	20.2	-22.3	Prostate cancer	+10	94.1	99.0	19.3
25.	Other malignant neoplasms	+1	-85.4	-20.4	-39.9	Conduct disorder	+1	-39.4	-38.0	0.2	Blindness and vision impairment	+2	19.9	22.9	-10.1
**FEMALES**															
**#**	**YLLs**	**change in rank**	**%** **change in counts**	**%** **change in all-age** **rates**	**% change in age-standar-dized rates**	**YLDs**	**change in rank**	**%** **change in counts**	**%** **change in all-age** **rates**	**% change in age-standar-dized rates**	**DALYs**	**change in rank**	**%** **change in counts**	**%** **change in all-age** **rates**	**% change in age-standar-dized rates**
1.	Ischemic heart disease	0	-39.5	-38.9	-64.0	Low back pain	0	24.7	25.9	0.1	Low back pain	+3	24.7	25.9	0.1
2.	Stroke	0	-32.2	-31.5	-57.5	Headache disorders	0	5.9	7.0	0.5	Ischemic heart disease	-1	-37.4	-36.8	-62.4
3.	Lung cancer	+8	123.7	125.9	56.3	Falls	0	16.7	17.8	-9.8	Headache disorders	+4	5.9	7.0	0.5
4.	Breast cancer	+1	11.9	13.0	-18.6	Neonatal disorders	0	-6.0	-5.0	2.5	Falls	+2	10.8	11.8	-15.6
5.	Alzheimer’s disease	+4	64.0	65.6	-13.5	Age-related hearing loss	0	37.6	38.9	-2.3	Stroke	-2	-22.7	-21.9	-50.8
6.	Colorectal cancer	+1	17.1	18.2	-20.0	Anxiety disorders	+1	5.5	6.5	0.3	Neonatal disorders	-4	-55.7	-55.3	-54.5
7.	Neonatal disorders	-4	-84.5	-84.4	-76.4	Diabetes	+3	51.2	52.7	12.5	Diabetes	+1	11.5	12.6	-18.6
8.	Congenital defects	-4	-79.3	-79.1	-70.2	Depressive disorders	0	11.2	12.3	-5.0	Lung cancer	+16	123.8	126.1	56.4
9.	Ovarian cancer	+6	26.6	27.9	-8.7	Neck pain	+2	25.4	26.6	0.4	Congenital defects	-4	-60.4	-60.0	-57.9
10.	Lower respiratory infect	-4	-14.5	-13.7	-49.9	COPD	-4	11.3	12.4	-22.3	Breast cancer	+1	16.2	17.4	-15.4
11.	Chronic liver disease	+10	49.5	51.0	21.9	Oral disorders	-2	9.7	10.8	-13.4	COPD	-1	13.0	14.1	-22.9
12.	Road injuries	-5	-57.8	-57.4	-56.6	Gynecological diseases	0	-3.5	-2.5	-5.0	Alzheimer’s disease	0	67.0	68.6	-12.0
13.	Cardiomypathy	0	18.2	19.4	-28.4	Blindness and vision impairment	0	22.9	24.2	-10.1	Age-related hearing loss	+2	37.6	38.9	-2.3
14.	Cervical cancer	-4	-33.8	-33.1	-49.9	Upper digest diseases	+1	-0.9	0.0	-10.0	Anxiety disorders	+2	5.5	6.5	0.3
15.	Pancreatic cancer	+8	33.3	34.6	-7.4	Stroke	-1	26.6	27.9	-17.0	Road injuries	-6	-31.9	-31.2	-40.0
16.	Self-harm	+2	-18.8	-18.0	-23.5	Congenital defects	+2	-2.2	-1.2	-1.2	Depressive disorders	+1	11.2	12.3	-5.0
17.	COPD	+2	16.5	17.6	-24.1	Road injuries	0	11.8	12.9	-7.2	Colorectal cancer	-3	18.0	19.2	-19.3
18.	Brain cancer	+6	5.5	6.6	-16.0	Asthma	+1	-4.0	-3.1	-8.3	Neck pain	+4	25.4	26.6	0.4
19.	Diabetes	-7	-32.9	-32.2	-56.0	Dermatitis	+1	-11.0	-10.1	1.1	Oral disorders	-1	9.7	10.8	-13.4
20.	Other malignant neoplasms	+2	-15.9	-15.0	-36.1	Bipolar disorder	+2	5.0	6.0	0.1	Upper digest diseases	+1	1.6	2.6	-12.6
21.	Stomach cancer	-5	-35.7	-35.1	-53.5	Schizophrenia	+3	15.9	17.0	3.6	Gynecological diseases	+2	-4.5	-3.5	-5.8
22.	Leukemia	+3	-11.8	-11.0	-35.7	Other mental disorders	+3	15.3	16.5	0.4	Blindness and vision impairment	+3	22.9	24.2	-10.1
23.	Gallbladder cancer	+5	-11.4	-10.5	-38.9	Alcohol use disorders	+5	18.2	19.3	9.7	Chronic liver diseases	+11	51.8	53.3	24.3
24.	Allcohol use disorders	+26	110.8	112.9	80.4	Osteoarthritis	+6	59.7	61.3	11.2	Ovarian cancer	+5	26.8	28.0	-8.4
25.	Uterine cancer	+7	36.4	37.8	-6.8	Epilepsy	+2	4.4	5.4	2.9	Lower respiratory infect	-12	-14.5	-13.7	-49.6

Colors indicate changes in ranks: redz = increase, green = decrease, and blue = no change

Color ranges were presented as follows

Decrease: <40 light green, 40–79 green, >79 dark green; Increase: <40 light red, 40–79 red, >79 dark red.

When looking at changes in ranks from 1990 to 2017 in Poland (Table 3), IHD remained the top cause of YLLs throughout this period. The largest increases among the top 10 causes were noted for chronic liver diseases (8 places), followed by colorectal cancer (4), lung cancer (3), self-harm (3), and Alzheimer’s disease (2), whereas decreases were seen for neonatal disorders (5), road injuries (2), stroke (1), and lower respiratory infections (1). Among the remaining top 25 causes, prostate and kidney cancer showed the greatest increase in ranks (16 and 14 places, respectively). Increases in ranks were also observed for alcohol use disorders (8 places); several cancers, including ovarian (10), pancreatic (6), brain (5), leukemia (5), breast (3), and other malignant neoplasms (2); and cardiomyopathy (1). Declines were seen for congenital defects (6 places) as well as stomach cancer (5), chronic obstructive pulmonary disease (COPD) (4), diabetes (3), and falls (1).

There were notable differences in YLL ranking and trends according to sex. Among males, the top five causes of YLLs were IHD, lung cancer, self-harm, stroke, and road injuries, whereas among females the order was IHD, stroke, lung cancer, breast cancer, and Alzheimer’s disease (Table 3). Road injuries were ranked 12^th^ for females and self-harm was ranked 16^th^. Furthermore, age-standardized rates for lung cancer decreased in males (29.6%) but increased substantially in females (56.3%). In males, both age-standardized and all-age rates increased for prostate cancer and chronic liver diseases. For both genders, all-age YLL rates decreased for IHD and stroke, despite population aging.

The trends observed in Poland were largely consistent with CE patterns S1A Fig. IHD was the leading cause of age-standardized YLLs in CE as a whole, and stroke was the second leading cause. However, alcohol use disorders ranked 11^th^ in Poland in 2017, but 20^th^ in the region. Conversely, hypertensive heart disease ranked 15^th^ in the region, whereas in Poland it decreased by 45% since 1990 and ranked 31^st^.

### Deviations from expected levels based on SDI

**Fig 2:**
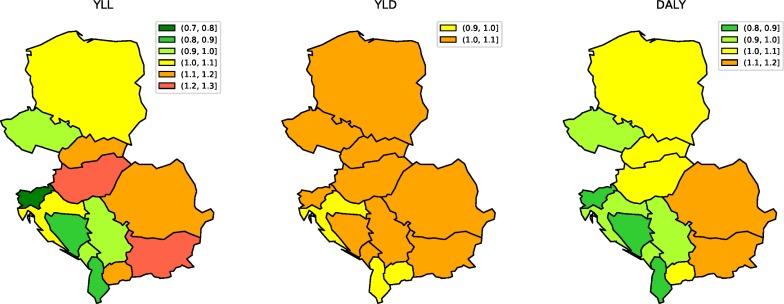
Observed-to-expected ratios for age-standardized YLL, YLD, and DALY rates for both sexes from all causes in Central European countries in 2017.

Fig 2 illustrates OER ratios for age-standardized YLL rates in CE countries in 2017. Significant differences were observed between countries, with Albania having the lowest (0.65) and Bulgaria the highest (1.10) ratio in the region. Of note, in 10 locations OERs were below 1.0; Poland’s OER was 0.96.

**Fig 3:**
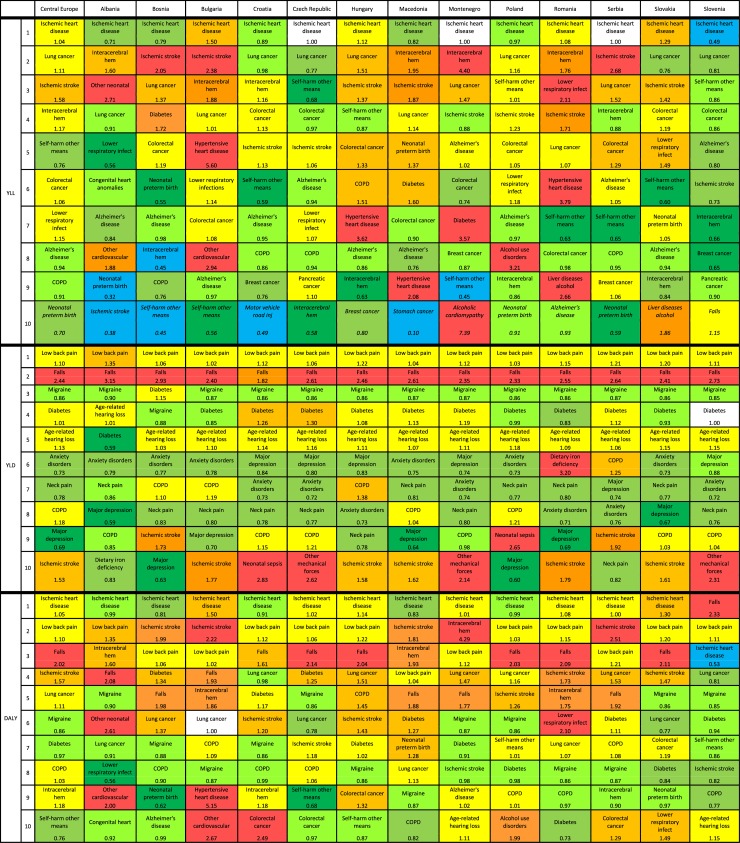
Leading Level 4 causes of YLLs (a), YLDs (b), and DALYs (c) in Central European countries, with the ratio of observed to expected (OER) age-standardized rates by location: (a) YLLs, (b) YLDs, (c) DALYs. Colors represent OER ranges: 0–0.54 = blue, 0.55–0.69 = green, 0.70–0.84 = light green, 0.85–0.99 = yellow green, 1.0 = white, 1.01–1.24 = dark yellow, 1.25–1.66 = orange, 1.67–2.91 = dark orange, 2.92+ = red.

In 2017, of the 10 leading causes of YLLs in CE, YLL rates were lower than expected for neonatal preterm birth complications (0.70), self-harm by other means (0.76), COPD (0.91), and Alzheimer’s disease (0.94) (Fig 3A). OERs were less than 1 for IHD in six CE countries; the lowest OER was observed in Slovenia (0.49). On the other hand, YLLs were much higher than expected for alcoholic cardiomyopathy in Montenegro (7.39); hypertensive heart disease in Bulgaria (5.60), Romania (3.79), and Hungary (3.62); intracerebral hemorrhage and diabetes in Montenegro (4.40 and 3.57, respectively); and alcohol use disorders in Poland (3.21).

### Years lived with disability (YLDs)

#### Overview

Between 1990 and 2017, the age-standardized all-cause YLD rate in Poland decreased by 4.0%, from 11,487 (95% UI: 8,581–14,864) to 11,033 (8,222–14,129) per 100,000 (Table 1, Fig 1). The rate declined by 2.6% in males and 5.2% in females. In CE as a whole, the rate decreased from 11,593 (8,678–14,879) to 11,099 (8,304–14,269) per 100,000 (4.3%) in the same period. Of all CE countries, only Romania and Hungary experienced greater reductions in YLD rates than Poland (6.9% and 6.4%, respectively). In 2017, the lowest rate in the region was observed in Albania (10,656; 7,965–13,666) and the highest in Bosnia and Hercegovina (11,368; 8,347–14,526). For comparison, overall age-standardized YLD rates in 2017 were 10,635 per 100,000, (8,012–13,711) in WE and 11,415 (8,855–14,736) in EE. However, the rank-order of the three regions varied by sex (Fig 1). In males, YLD rates were lowest in WE (9,936; 7,486–12,852) followed by EE (11,095; 8,320–14,306) and CE (11,228; 8,489–14,446), whereas in females, the rates were lowest in CE (10,952; 8,177–14,163), followed by WE (11,351; 8,516–14,645) and EE (and 11,714; 8,777–15,079).

#### Main causes of YLDs

In 2017, CMNN causes contributed 5.7% (95% UI: 4.5–7.1) to all-age YLDs in Poland, NCDs contributed 78.2% (75.9–80.2), and injuries 16.1% (14.4–18.1) (Table 2). Of note, while a significant decline for CMNN-attributed YLDs was observed from 1990 to 2017 (22.7%, from 1,048.4 to 810.2 per 100,000), it was accompanied by increases for NCDs (21.5%, from 9,179.5 to 11,154.3) and injuries (14.6%, from 2,010.2 to 2,303.1) in the same period. In CE, CMNN causes contributed 5.8% (4.9–7.0) to total YLDs, NCDs contributed 78.5% (76.3–80.6), and injuries 15.7% (14.0–17.6). All-age YLD rates for CMNN causes fell by 20.5%, from 1,055.4 per 100,000 in 1990 to 839.3 in 2017, whereas those from NCDs increased by 19.5%, from 9,508.9 to 11,364.0 and those from injuries by 15.7%, from 2,047.2 to 2,270.7. YLD rates for Level 2 causes (22 groups of conditions) are provided in the S2 Table.

In 2017, the top 10 Level 3 causes of age-standardized YLDs in Poland were low back pain, falls, headache disorders, neonatal disorders, diabetes, age-related hearing loss, depressive disorders, anxiety disorders, neck pain, and COPD (Table 3). Between 1990 and 2017, changes in the rank-order of the top five conditions were minimal. Among the top 25 causes of YLDs, declines in age-standardized rates were observed for 15; however, the declines did not exceed 10%, except for COPD (24.5%), road injuries (14.8%), stroke (13.2%), oral disorders (12.8%), asthma (11.4%), and blindness and vision impairment (10.1%). Diabetes, drug use, and alcohol use disorders showed the largest increases in rates (21.2%, 11.0%, and 9.3%, respectively). The greatest differences between change in all-age and age-standardized rates (30% or greater) were noted for diabetes, osteoarthritis, and stroke, indicating that these diseases largely affect the elderly and, therefore, become more prominent causes of YLDs in an ageing population.

While the top four causes of YLDs were the same in males and females, ranking of the conditions differed (Table 3). Other sex differences included much higher ranking of anxiety disorders in females, and alcohol use disorders and road injuries in males. Between 1990 and 2017, age-standardized YLD rates for Polish males increased for diabetes (28.6%), drug (11.6%) and alcohol (8.6%) use disorders, neonatal disorders (10.7%), depressive disorders (5.3%), and low back pain (2.6%). For females, rates increased for diabetes (12.5%), osteoarthritis (11.2%), alcohol use disorders (9.7%), schizophrenia (3.6%), and epilepsy (2.9%). In terms of all-age YLDs in males, the highest increase was observed for diabetes (79.2%), followed by osteoarthritis, urinary diseases, male infertility, stroke, other cardio-vascular diseases, and age-related hearing loss (35%–67%). In females, YLDs due to the latter three causes increased by 27%–39%, while YLDs due to Alzheimer’s disease increased by 79.9% and those due to osteoarthritis by 61.3% in the same period.

Country-level data revealed that in 2017 low back pain ranked first in age-standardized YLD rates in CE, followed by falls, headache disorders, and neonatal disorders S1b Fig. The pattern has not changed when compared to 1990. The main contributors to age-standardized YLDs in CE were comparable to those reported for Poland.

#### Deviations from expected levels based on SDI

In 2017, all CE countries had OERs for all-cause YLDs above or equal to 1 (1.0–1.07), with Albania having the lowest OER and the Czech Republic, Hungary, Romania, Bosnia and Herzegovina, and Slovenia tied for the highest (Fig 2). Fig 3B shows the OERs for the top 10 causes of YLDs for each country in 2017. All CE countries had higher than expected YLDs from falls, with OER greater than 2 for all countries but Croatia. YLDs due to iron deficiency were more than triple the expected rate in Romania, while Poland and Croatia had higher than expected rates of neonatal sepsis (2.65 and 2.83, respectively). The OER for injuries due to other mechanical forces was more than 2 in Montenegro (2.14), Slovenia (2.31), and Czech Republic (2.62). OERs for low back pain and age-related hearing loss were slightly higher than expected in all CE countries, and the rates for stroke were higher than expected in seven countries. Of note, OERs for major depression were low in the region (0.59–0.88), with the lowest ratio observed in Albania. YLDs due to anxiety disorders, neck pain, and migraine were lower than expected in all CE countries.

### Disability-adjusted life years (DALYs)

#### Overview

**Fig 4:**
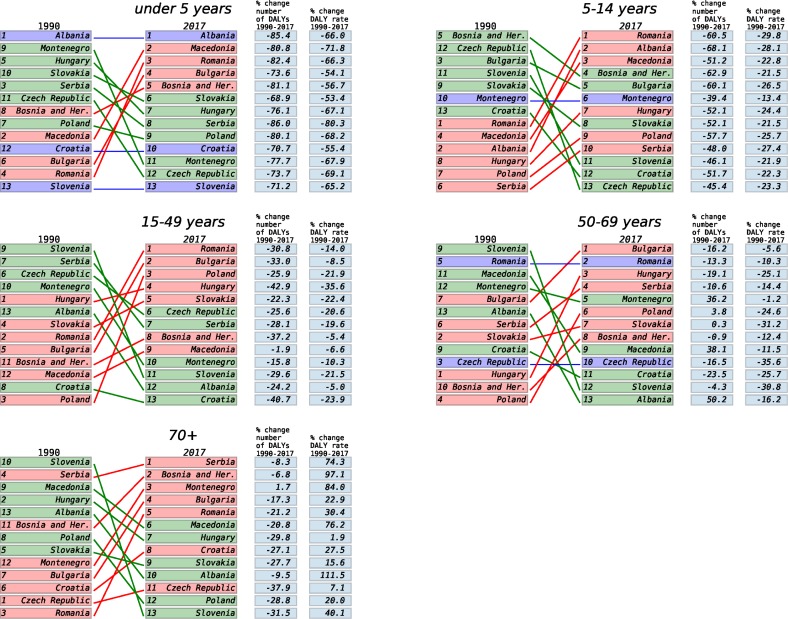
Ranking of Central European countries according to all-cause DALY rates for both sexes combined in 1990 and 2017, by age categories.

Between 1990 and 2017, the age-standardized DALY rate in Poland decreased by 31.7%, from 33,741 per 100,000 (95% UI: 30,817–37,080) in 1990 to 23,040 (20,212–26,249) in 2017 (Table 1). The rate decreased by 33.4% in males and 29.8% in females (Fig 1). The all-age DALY rates fell by 8.2% in the same period S1a Table. In the CE region as a whole, the age-standardized DALY rate decreased by 30.2%, 31.5% in males and 28.7% in females (Fig 1). CE countries with the lowest rates in 1990 were Montenegro, Slovenia, and Bosnia and Hercegovina, whereas in 2017, the lowest rates were in Slovenia, the Czech Republic, and Croatia. Poland improved significantly in DALY ranking, from ninth to fifth best, a greater improvement than any other CE country except the Czech Republic (from seventh to second) and Slovakia (from 10^th^ to sixth). The rise in ranking for Poland was especially strong for the group 70 years of age and older (sixth to second), as shown in Fig 4. However, the DALY rate decreased the most (68.2%) in the group <5 years of age. In all other age groups, DALY rates decreased by 22%–29%.

In WE, meanwhile, the age-standardized DALY rate decreased by 25.3%, from 24,828 (22,169–28,000) to 18,536 (15,928–21,551), 30.6% in males and 20.0% in females. In EE the rate was higher, at 36,827 (33,891–40,128) in 1990, and dropped to 32,411 (29,593–35,640) in 2017, i.e., 12.0% overall, 11.5% in males and 13.7% in females (Fig 1).

#### Main causes of DALYs

CMNN causes contributed 4.8% (4.3–5.4) to total all-age DALYs in Poland in 2017, NCDs contributed 82.4% (81.3–83.5), and injuries 12.7% (11.8–13.9) (Table 2). All-age DALY rates for CMNN causes fell by 48.2%, from 3,044.8 per 100,000 in 1990 to 1,577.4 in 2017, whereas those from NCDs fell by 17.8%, from 27,527.7 to 26,980.0, and those from injuries by 2.0%, from 5,088.6 to 4.184.2. In CE, CMNN causes contributed 4.8% (4.4–5.2) to total DALYs, NCDs 83.9% (82.7–84.9), and injuries 11.3% (10.4–12.4). All-age DALY rates for CMNN causes decreased by 51.6%, from 3,461.8 in 1990 to 1,674.1 in 2017, whereas those from NCDs increased by 2.1%, from 28,576.8 to 29,185.8, and those from injuries decreased by 22.7%, from 5,108.3 to 3,948.4. DALY rates for Level 2 causes are provided in S2 Table.

As shown in Table 3, among the top 25 Level 3 causes, all-age DALY rates increased for 18; the greatest increases were observed for chronic liver diseases (75.0%) and Alzheimer’s disease (70.7%). Significant increases were also observed for colorectal cancer (42.0%) and alcohol use disorders (41.4%). The difference between changes in all-age and age-standardized rates between 1990 and 2017 was 30% or greater for all abovementioned causes except alcohol use disorders.

In 2017, the top three causes of age-standardized DALY rates in Poland were IHD, low back pain, and falls, even though the rates for IHD and falls declined (62.2% and 13.0%, respectively) (Table 3). Stroke, neonatal disorders, lung cancer, headache disorders, road injuries, diabetes, and self-harm were the other top 10 causes of DALYs. Except low back pain, headache disorders, and self-harm, for which very slight (0.02%–1.3%) increases were observed, all these conditions showed a reduction in age-standardized DALY rates (5.0%–62.2%). Stroke, neonatal disorders, and road injuries declined (i.e., improved) in ranking, while the remaining top 10 causes (except IHD) increased (Table 3). Among other conditions, large reductions in age-standardized DALY rates (36%–58%) were noted for congenital defects, COPD, and lower respiratory infections. Alcohol use disorders increased substantially in ranking (from 17th to 12th) as did chronic liver diseases (from 23rd to 14th), with an increase in age-standardized rates of 20.2% and 36.2%, respectively. By contrast, colorectal cancer, age-related hearing loss, and blindness and vision impairment increased in ranks, even though their age-standardized rates declined (2.9%–10.1%). There were also increases in ranks with slight decreases in rates (0.01%–0.9%) for a small number of other causes, notably anxiety and depressive disorders. In general, between 1990 and 2017, of the top 25 causes of age-standardized DALY rates, declines were observed for all but six; in three of those the increase did not exceed 1%.

As Table 3 and S1a Fig show, there was a significant overlap between the top 25 causes of DALYs in Poland and the CE region in 2017. Of note, the top nine causes of DALYs in Poland and CE were the same, albeit in a slightly different order. However, alcohol use disorders ranked 12th in Poland and 19th in the region.

Table 4 displays country rankings, organized from best (lowest) to worst, according to age- standardized DALY rates for the top 25 causes in Poland and other CE countries, for both sexes combined, in 1990 and 2017. In 2017, Poland ranked first for depressive disorders, third for IHD and stroke, fourth for low back pain and other cardiovascular disorders, fifth for diabetes, and sixth for COPD and Alzheimer’s disease. On the other hand, Poland ranked 13^th^ (worst) for self-harm, alcohol use disorders, and upper digestive system diseases, and 12^th^ for road injuries, headache disorders, and anxiety disorders. Between 1990 and 2017, Poland’s ranking among 13 CE countries improved for 11 of the top-25 causes of DALYs and worsened for 10.

**Table 4:** Ranking of CE countries according to age-standardized DALY rates, for both sexes combined, in 1990 and 2017.

Cause	Country																									
	Albania		Bosnia		Bulgaria		Croatia		Czech Repub.		Hungary		Macedonia		Montenegro		Poland		Romania		Serbia		Slovakia		Slovenia	
Year	1990	2017	1990	2017	1990	2017	1990	2017	1990	2017	1990	2017	1990	2017	1990	2017	1990	2017	1990	2017	1990	2017	1990	2017	1990	2016
Ischemic heart disease	2	6	6	7	10	13	5	2	12	4	9	9	4	5	3	8	**11**	**3**	8	10	7	12	13	11	1	1
Low back pain	11	10	1	1	3	3	6	9	9	6	13	12	2	2	5	5	**4**	**4**	10	7	7	8	12	13	8	11
Stroke	4	7	5	8	11	10	6	6	7	2	8	5	13	12	10	13	**2**	**3**	9	9	12	11	3	4	1	1
Falls	4	2	5	3	7	7	6	1	11	12	13	9	1	5	3	4	**8**	**10**	12	8	2	6	10	11	9	13
Lower respiratory infect.	13	12	1	3	11	11	2	1	4	8	5	2	10	6	6	5	**7**	**9**	12	13	8	7	9	10	3	4
Road injuries	4	9	1	2	6	11	8	10	5	7	10	1	2	4	3	8	**12**	**12**	9	13	7	3	11	5	13	6
Lung cancer	1	1	5	9	3	5	7	6	12	3	13	13	2	8	9	12	**11**	**10**	4	7	10	11	8	2	6	4
Neonatal disorders	9	12	11	10	2	8	5	6	3	1	7	4	13	13	10	5	**8**	**7**	4	9	12	11	6	3	1	2
Age-related hearing loss	13	13	12	12	8	8	4	5	2	2	3	4	11	11	6	9	**9**	**7**	7	6	10	10	5	3	1	1
Congenital birth defects	3	13	2	11	9	12	4	5	5	2	8	6	11	8	1	1	**10**	**7**	12	10	13	4	7	9	6	3
Headache disorders	1	13	3	4	9	8	13	10	8	2	7	11	6	3	12	9	**10**	**12**	2	5	4	6	11	7	5	1
Chronic liver diseases	3	2	7	5	6	10	9	7	8	6	13	11	2	3	1	1	**4**	**9**	10	13	5	4	11	12	12	8
Self-harm	2	2	3	9	6	4	10	6	9	8	13	11	1	1	5	7	**7**	**13**	4	3	11	10	8	5	12	12
COPD	7	3	11	10	6	11	3	7	4	8	12	13	8	5	1	1	**10**	**6**	13	9	9	12	2	4	5	2
Diabetes	1	1	8	13	6	6	9	8	7	9	10	7	11	12	12	10	**5**	**5**	2	2	13	11	4	4	3	3
Other cardiovascular	7	12	12	11	13	13	10	1	8	10	4	3	6	7	1	5	**9**	**4**	5	6	3	8	11	9	2	2
Colorectal cancer	1	1	5	7	6	8	8	10	13	6	12	13	2	3	3	2	**9**	**9**	4	5	10	11	11	12	7	4
Depressive disorders	1	2	7	3	8	7	11	12	10	10	12	11	4	4	6	8	**2**	**1**	3	6	9	9	5	5	13	13
Cardiomyopathy	8	6	5	7	1	2	4	3	2	1	11	10	6	8	10	12	**9**	**11**	12	13	7	9	3	4	13	5
Stomach cancer	4	12	3	10	8	9	13	7	6	1	10	5	12	13	1	2	**9**	**8**	5	11	2	6	7	4	11	3
Alcohol use disorders	1	1	12	10	5	3	9	7	4	8	13	9	2	2	3	4	**11**	**13**	10	5	6	6	8	11	7	12
Alzheimer’s disease	1	7	2	12	11	10	12	8	9	4	4	3	3	2	10	11	**13**	**6**	7	9	5	13	8	5	6	1
Anxiety disorders	2	7	5	5	13	13	12	9	7	3	6	11	3	4	8	8	**9**	**12**	1	1	4	6	11	10	10	2
Upper digestive system diseases	8	5	10	6	5	9	1	1	4	4	11	10	3	3	2	7	**12**	**13**	13	12	7	8	9	11	6	2
Neck pain	6	13	11	2	8	12	12	11	4	3	2	7	9	4	13	10	**10**	**9**	1	8	7	6	5	5	3	1

Poland is shaded and shown in bold. Colors indicate changes in ranks: red = increase, green = decrease, and white = no change

#### Deviations from expected levels based on SDI

As shown in Fig 2, the OERs for DALYs in the CE countries were generally around or below 1; Albania had the lowest OER (0.77), whereas the OER for Poland was 1.0. In four countries, Hungary, Slovakia, Bulgaria, and Romania, observed DALY rates were slightly worse than expected (1.01–1.07). Fig 3C shows the top 10 causes of DALYs in CE and the variation from expected values based on SDI. Observed DALYs for IHD were higher than expected in six countries: Bulgaria, Slovakia, Hungary, Romania, the Czech Republic, and Montenegro. For low back pain and falls, observed DALYs were greater than expected in all CE countries, with the highest OERs observed for low back pain in Albania (1.35) and for falls in Slovenia (2.33). For the remaining causes, strong country-specific patterns were observed. The OER for ischemic stroke in CE was 1.57, with the highest observed OER in Serbia (2.51) and the lowest in Slovenia (0.82). Particularly high OERs were observed for hypertensive heart disease in Bulgaria (5.15) and for intracerebral hemorrhage in Montenegro (4.29).

## Discussion

### Overview of the results

Among the CE countries, the lowest age-standardized DALY and YLL rates in 2017 were achieved by Slovenia, followed by the Czech Republic, whereas the highest rates were seen in Romania and Bulgaria. Age-standardized DALY and YLL rates in the CE region were substantially higher than the rates in the WE region throughout the study period. However, the difference was smaller for females compared with males and was diminishing over time, especially for females. In fact, the DALY and YLL rates for females in the top-performing CE country in 2017 were lower than the corresponding WE rates. Furthermore, the age-standardized YLD rates for females in the CE region were lower than the corresponding rates in the WE region. DALY rates for the CE countries correlated moderately with SDI values; however, some countries performed much better than expected (e.g., Albania, Slovenia, Macedonia) while others performed worse than expected (e.g., Serbia, Bulgaria, Romania, Slovakia). The reasons for this variation are probably complex and may involve economic, cultural, historical, and geographical factors as well as differences in health systems and policies.

Between 1990 and 2017, age-standardized YLL rates for all causes declined in Poland by 46.0%, whereas YLDs declined by 4.0%, and DALYs by 31.7%. These trends were largely consistent with CE trends for all three metrics. However, Poland performed better than CE in general; only three out of 12 CE countries achieved a greater reduction in age-standardized YLL rates, and two countries achieved a greater reduction in YLD rates. Poland ranked fifth best for DALYs and, along with Slovakia, experienced greater improvements in ranking than any other CE country except the Czech Republic.

The top three causes of premature deaths in Poland in 2017 were IHD, lung cancer, and stroke, whereas the leading causes of disability were lower back pain, falls, and headache disorders. Of the top 25 causes of premature mortality, only eight appeared in the top 25 causes of disability. IHD, lower back pain, and falls were the top-ranking causes of DALYs. Two causes, diabetes and self-harm, appeared among the 10 leading causes of DALYs in 2017, though not in 1990. Of the 25 most important causes of YLLs and DALYs, IHD, neonatal disorders, and congenital defects showed the largest decrease in age-standardized rates from 1990 to 2017, followed by road injuries and stroke, while alarming increases for alcohol use disorders and chronic liver diseases were observed for both metrics.

Of the top 25 causes of YLLs and YLDs in Poland in 2017, all but three were among the top 25 in CE in the same year. The ratio of DALYs from NCDs to those from CMNN diseases was 17.1 in 2017, compared with 9.0 in 1990, showing a strong epidemiological transition. Poland had a ratio of observed to expected YLL, YLD, and DALY rates of about 1.0 in 2017; the worst performance relative to SDI-based expectations was noted for alcohol use disorders for YLLs, neonatal sepsis and falls for YLDs, and alcohol use disorders and falls for DALYs [25].

### Interpretation of trends for major conditions

Age-standardized YLL rates from IHD and stroke have been declining in high-income countries for several decades, [10] and Poland followed this trend over the past 25 years. Factors that may have contributed to the massive declines in death rates from cardiovascular and cerebrovascular diseases [25] include changes in risk factors, including dietary habits, better blood pressure control, improved quality of health services, and adequate access to modern treatment methods, especially pre-hospital emergency care and cardiac surgery (e.g., the POLCARD 2013–2016 program) [26–29]. However, some high-income countries, notably the US, have recently experienced a flattening and possibly a reversal of this long-term trend [24]. This may be associated with the epidemic of obesity and sedentary lifestyle as well as other factors. To avoid similar developments in Poland, it is paramount that public health efforts to further reduce the risk factors for cardiovascular diseases continue and receive strong government support [30].

Low back pain is the second most important cause of DALYs in Poland and one of the leading causes of disability in all CE countries. The economic impact of this condition, due to reduced productivity, is also enormous [31]. Close to 40% of low back pain can be attributed to known risk factors, such as occupational risks or increased body mass index [11]. While insufficient knowledge of risk factors hinders implementation of preventive strategies for this condition, ergonomic measures, obesity reduction, physical therapy, analgesic medication, and in some cases surgery can be effective in reducing pain and improving function [32].

The age-standardized rate for falls has declined slightly, but the all-age DALYs increased due to population aging; rates were greater than expected in Poland, as well as in all other CE countries. Multimodal falls prevention programs which target a combination of risk factors, including prevention of and screening for osteoporosis, sarcopenia and frailty, and are tailored toward selected high-risk groups, should be implemented or expanded to reduce fall-related injuries [33–35]. Such programs, carried out by the European Innovation Partnership on Active and Healthy Ageing (EIP AHA), are good examples to learn from [34].

Between 1995 and 2015, infant mortality among preterm births decreased more than three-fold (128.5 to 36.8 per 1,000 preterm live births), whereas early neonatal mortality decreased almost four-fold [36]. This shows how programs focused on CMNN, such as universal access to antenatal care for early detection of complications of pregnancy, scrupulous newborn screening for congenital birth defects, and adequate neonatal vaccination policies, accompanied by parental education [3, 36] could potentially engender dramatic reductions in DALYs, especially in the under-5 age group. However, neonatal disorders are still the fifth leading cause of DALYs in Poland, partly because the decrease in infant and neonatal mortality among preterm births resulted in longer-lasting disability [36].

Cancer generally causes relatively few YLDs (except in terminal stages) but is a very important cause of YLLs. The most DALYs due to cancer in Poland are caused by cancer of the lung (ranked sixth overall), followed by colon/rectum, breast, stomach, pancreas, and brain. Although age-standardized rates for all major cancers have declined, all-age rates for most cancers (e.g., lung, colorectal, breast, pancreas, prostate, brain) are increasing due to population aging. Therefore, continued efforts to reduce cancer burden are needed and should include a combination of primary prevention focused on established risk factors (smoking, diet, alcohol, obesity, physical activity, occupational risks, and other factors), screening, and access to modern medical, surgical, and radiation treatment [37].

Prevention and treatment of headaches, specifically migraine, remains a challenge, and more research into risk factors and underlying biological mechanisms is warranted. Migraine should receive special attention not only because of the high burden due to its prevalence and associated disability, but also due to a potential association with increased risks of myocardial infarction, stroke, venous thromboembolism, and atrial fibrillation or flutter [38].

DALYs due to road injuries have decreased considerably, likely due to better vehicle safety, more rigorous regulations and law enforcement, and improvements in the road network, particularly in the number of motorways [39]. However, Poland ranked second worst in CE in terms of DALY rates from road injuries and has the highest number of deaths per billion vehicle-km [40]. Consequently, intervention priorities, such as pedestrian protection and speed management, were defined in the Polish National Program for Road Safety for 2018–2019. Hungary, Slovakia, and Slovenia, the recent recipients of the European Transport Safety Council road safety awards, are examples to follow in terms of road safety improvements in the CE region [41]. Potentially effective measures include mandatory road safety impact assessment of road infrastructure projects, road safety audit by an independent team, vehicle owner liability for drunk driving and speeding, and higher penalty points and fines, with regulations enabling more effective enforcement.

Diabetes (mostly type 2) contributes substantially to both YLLs and YLDs and although the age-adjusted DALY rate has declined somewhat compared to 1990, a slightly increasing trend was noted in the last decade [42]. Due to its strong relationship with obesity and sedentary lifestyle, prevalence of type 2 diabetes is unlikely to decrease in the foreseeable future. At the population level, it is well documented that implementation of early screening strategies for identification of individuals at risk (including those with prediabetes) may significantly reduce the prevalence of diabetes, overall costs of treatment of conditions associated with hyperglycemia, and premature deaths [42, 43]. Early detection and treatment of diabetes in Poland and other CE countries could be facilitated by improvements in diabetes surveillance systems, including the development of diabetes registries [42].

Poland is ranked worst in CE in terms of DALY rates from self-harm and experienced a significant worsening in ranking since 1990. Furthermore, suicide rates remained fairly constant since 1990, whereas CE as a whole has seen a gradual improvement. Therefore, measures that reduce the incidence of suicide, listed in the Polish National Program for the Preservation of Mental Health for 2017–2022, e.g., prevention, early detection, and treatment of depression, creation of suicide prevention centers, or enhanced training in suicide prevention for professionals and community volunteers, should be integrated with the existing methods of assessing and managing suicide risk [44]. It should be noted that depressive and anxiety disorders are important contributors to DALYs regardless of their potential link with self-harm. Although the burden of depression is currently smaller in Poland than in other CE countries, it is likely to increase if Poland follows the trend observed in WE and other high-income countries [45]. Therefore, current efforts to reduce stigma of mental illness, improve access to professional treatment and, most importantly, further embed a range of preventive strategies oriented toward high-risk groups in primary care [3, 44, 45] should be expanded.

For alcohol use disorders, the current DALY rate in Poland is much higher than in any other CE country and higher than in WE as a whole (albeit lower than in EE countries) [10]. In addition, YLLs for this cause are three times higher than expected. An increase in DALY rates occurred mainly in the period 2000–2007; a slight decline was observed in the last decade. Of note, alcohol consumption in Poland (similar to some Northern and Eastern European countries) differs from the Western European pattern, as Poles tend to drink hard liquor (e.g., vodka) and beer, rather than wine, and tend to engage in binge drinking more frequently [3, 46]. The large burden of alcohol use disorders urgently requires a policy response (e.g., taxation and restrictions on availability and marketing) and a health system response (expansion of treatment) [47, 48].

Alzheimer’s disease and osteoarthritis are ranked outside of the top 10 causes of DALYs and represent different categories of diseases, but have some common features. They are very common, strongly related to age, can be severely disabling, and are difficult to prevent or treat effectively, although loss of weight can help prevent osteoarthritis and joint replacement is effective for advanced disease. These two conditions are important because they are responsible for a significant proportion of DALYs and are unlikely to diminish in the foreseeable future.

Over the past 27 years in Poland, the conditions that demonstrated the largest drop in DALY rates and ranking are lower respiratory infections and COPD. For COPD, the most important risk factor is smoking, and a reduction in COPD is mostly a result of long-term decreasing trends in smoking rates. A similar trend has been observed elsewhere [49].

### Socioeconomic changes and epidemiological transition

Factors that might have affected the study findings are likely complex. Although it is difficult to identify the specific causes of the differences in YLLs, YLDs and DALYs among the CE countries, differences in political systems, socioeconomic conditions, health behaviors (e.g., smoking, alcohol, diet), and health care systems are likely to play a role [50]. Changes in population health have paralleled rapid socio economic development that took place in these countries over the past quarter of a century and can be, at least partly, attributed to it [7, 8, 51, 52]. SDI in Poland improved from 0.66 in 1990 to 0.84 in 2017, compared with increases from 0.66 to 0.81 in the CE region, 0.68 to 0.79 in EE, and 0.76 to 0.86 in WE. Poland ranked third for SDI in CE in 2017 and second (after Bosnia and Herzegovina) for relative improvement in SDI between 1990 and 2017.

There have been substantial between-country differences in CE in terms of national income and health expenditures (HE) [53]. In Poland, HE per capita increased from US$ 197 in 1995 to 809 in 2016 [54]. As a result, Poland improved its position from 6^th^ to 4^th^ during this period. Greater relative improvements were seen in Bosnia and Herzegovina (US$ 44.6 to 444) and Romania (US$ 53 to 476) [2, 55]. However, as a percentage of the GDP, HE in Poland in 2016 ranked third lowest among the CE countries [5]. Plans to increase the health budget in the next decade have been announced [56]. Nonetheless, improvements in health care and public health programs, in addition to changes in the distribution of risk factors, likely have played an important role in reducing disease burden. In particular, a dramatic drop in neonatal and infant deaths can be partly explained by substantial improvements in prenatal and postpartum care over the past two decades [57].

Detailed analyses of changes in DALY rates in Poland and other CE countries provide insights into public health successes and areas where some countries might be falling behind. This, in turn, could help galvanize efforts aimed at minimizing the gap in disease burden between CE and WE. One-third of the decline in age-standardized DALYs in Poland between 1990 and 2017 was due to CMNN causes. All-age DALY rates for CMNN causes decreased by half, whereas injuries and especially NCDs decreased to a lesser extent. A similar trend was seen in other CE countries. In Poland and CE as a whole, NCDs are now responsible for much more health loss due to premature death or disability than injuries and CMNN diseases. The continuous increase in the contribution of NCDs to total disease burden in Poland and CE mimics the epidemiological transition taking place globally [12, 58, 59]. However, differences between locations were observed for the ratios of DALYs from NCDs to those from CMNN diseases in 1990 and 2017. The rising problem of NCDs poses challenges for the Polish health system that need to be addressed. Recently implemented national programs for cancer, cardiovascular diseases, mental health, as well as the promotion of healthy and active aging [25, 34, 60–63] and the Health Needs Map project for 30 groups of diseases [64] are positive developments in this area.

The shift from acute diseases to chronic disability is associated with the need for qualified health personnel. However, since joining the European Union, Poland has faced a dramatic shortage of physicians (mainly specialists) and nurses, who often seek jobs in other EU countries, as well as ongoing workforce aging [65]. These are important obstacles to strengthening the health care system. The Polish government has already identified training of health professionals as a top priority, but their retention needs system changes which are challenging to implement.

### Health inequalities

Poland (and CE in general) is characterized by a large difference in age-standardized YLLs and DALYs between males and females, with males experiencing much higher YLL rates for all leading causes of death. The male to female ratio for DALYs in Poland decreased only slightly, from 1.6 in 1990 to 1.5 in 2017, compared to a drop from 1.3 to 1.2 in WE. Also, greater relative declines in YLLs and YLDs were observed for females than males during this period. In terms of non-fatal health outcomes, all-cause YLD rates in males and females are similar, although there are large differences for some conditions. Differences in premature mortality between males and females can be partially explained by differences in the distribution of risk factors (e.g., smoking, alcohol, diet, a lack of physical activity and occupational exposures) for major categories of chronic conditions, such as CVD and cancer [66, 67]. In addition, men are less likely to participate in screening programs [67]. Other factors, including biopsychological differences, may also be important, especially for mental health and self-harm [7]. The YLL rate for self-harm in Poland is almost eightfold higher in males. The male/female mortality gap is a serious concern that, in our view, has not received sufficient attention, and few policies explicitly designed to reduce this gap have been implemented. There also exist substantial disparities in health between the provinces, rural versus urban areas, and socioeconomic groups within Poland and other CE countries [3], but such data are currently not available from the GBD.

### Limitations

Some important limitations of this study should be recognized. First of all, our results share the limitations of GBD data in general, as described in detail in GBD publications [9–12, 14, 15, 58, 59]. They include variation across locations in cause of death certification and reporting, insufficient data on disease prevalence and severity distribution, methodological issues around disease definitions, modeling of comorbidity, and accuracy of disability weights assigned to different diseases. Second, although the quality of data in Poland has improved compared with 1990 [3, 10], the existing information systems have important gaps that limit their ability to monitor population health and potential health threats. According to the Polish Office of Health Statistics (GUS), the proportion of ill-defined causes of deaths in Poland is very high and on the rise throughout the last decade [68]. In 2016, of the total of 388,009 deaths, causes assigned for 113,322 (29.2%) were considered “garbage codes” (codes that should not be used for the underlying cause of death) by the GBD. These garbage codes influence the quality of information on the causes of death, which may hinder the identification of health priorities and planning of health interventions [69]. Improving the quality of cause of death certification is possible through training of physicians, regulatory measures, and technological innovation. A good example of the latter is a recent effort to introduce electronic death certification, combined with online support and automated verification of the code [70]. In our view, this issue should be considered an urgent priority by the health care administrators in every country where the “garbage code” problem is substantial. Third, there is a lack of adequate information regarding data generated by primary and specialist care which are related to less dramatic health events than hospitalization or death [3]. A pilot program to address this data gap, involving 100 primary care units, has been recently introduced [71]. Fourth, in assessing factors that may have influenced changes in disease burden we have not been able to take into account migration, both within Europe and between CE and countries on other continents. For Poland and most CE countries, the number of immigrants from the Middle East and North Africa has been relatively low. However, over the past two decades Poland has experienced high levels of immigration (primarily from Ukraine) and emigration (primarily to the UK, Germany and other EU countries). Similar—although lower–migration into and out of the country was observed between 2000 and 2011 in Romania [72]. Finally, current data do not consider variation in health indicators across regions (provinces) within each CE country. To address this limitation, Poland has recently initiated a collaboration with the GBD to generate subnational estimates of disease burden.

## Conclusions

Health in Poland is improving, and the country outperforms CE as a whole for YLLs, YLDs, and DALYs. This is a result of a faster decrease in age-standardized YLL rates between 1990 and 2017 compared with CE, accompanied by faster, however more modest, declines in YLDs. As a result, the all-cause age-standardized DALY rate decreased by about a third. Poland has made notable improvements in preventing child deaths, cardiovascular diseases, and road injuries. On the other hand, there are worrisome trends in alcohol use disorders, chronic liver disease, diabetes, and self-harm. The shift to chronic disability, together with marked between-gender health inequalities, poses a challenge for the healthcare system, particularly in light of a significant shortage and aging of the workforce and relatively low public health expenditures.

Geographical variation in leading causes of premature death and disability in CE is fairly limited, although some between-country differences exist. Health policies and practices in good regional performers offer examples that can be followed by other countries in order to reduce disease burden. To minimize the gap between CE and WE, an integrated response, which addresses the causes of death and ill-health, particularly those for which rates have increased and higher-than-expected rates are observed, is needed in all CE countries.

## Supporting information

S1 Table Estimates of all-age YLLs, YLDs, and DALYs rates (95% UI) and country ranks (from best to worst) for Poland and other Central European countries, for both sexes combined, males and females, in 1990 and 2017. (DOCX)

S2 Table All-age rates, percentage contribution, and relative (%) change for Level 2 causes of YLLs, YLDs, and DALYs for Poland and Central Europe, both sexes combined, in 1990 and 2017. (DOCX)

S1 Fig Top 25 Level 3 causes of YLLs (a), YLDs (b), and DALYs (c) in the CE region for both sexes combined in 1990 and 2017: (a) YLLs, (b) YLDs, (c) DALYs. Conditions are ranked according to age-standardized rates, from highest to lowest. Colors indicate changes in rank: red = increase, green = decrease, and purple = no change. The numbers are percentage changes in counts, all-age rates, and age-standardized rates.
(DOCX)
