# Supplementary material for: Changes in disease burden in Poland between 1990–2017 in comparison with other Central European countries: A systematic analysis for the Global Burden of Disease Study 2017
Source: PLoS One. 2020 Mar 2;15(3):e0226766. doi: 10.1371/journal.pone.0226766 (PMC7051048; doi:10.1371/journal.pone.0226766)
Supplement: S1 Table — (DOCX) [file pone.0226766.s001.docx]

**Appendix**

S1 Table. Estimates of all-age YLLs, YLDs, and DALYs rates (95% UI) and country ranks (from best to worst) for Poland and other Central European countries, for both sexes combined, males and females, in 1990 and 2017

| **Country** | **All-Age YLL rate**  **per 100,000** | | | | **All-Age YLD rate**  **per 100,000** | | | | **All-Age DALY rate**  **per 100,000** | | | |
| --- | --- | --- | --- | --- | --- | --- | --- | --- | --- | --- | --- | --- |
|  | **1990** | | **2017** | | **1990** | | **2017** | | **1990** | | **2017** | |
|  | Rate  95% UI | Rank | Rate  95% UI | Rank | Rate  95% UI | Rank | Rate  95% UI | Rank | Rate  95% UI | Rank | Rate  95% UI | Rank |
| **Total** | | | | | | | | | | | | |
| **Central Europe** | **24,535** |  | **20,335** |  | **12,611** |  | **14,474** |  | **37,147** |  | **34,808** |  |
|  | **24,482-24,589** |  | **19,966-20,701** |  | **9,444-16,220** |  | **10,868-18,600** |  | **33,956-40,475** |  | **31,139-38,951** |  |
| Albania | 18,832 | 3 | 15,194 | 2 | 9,760 | 1 | 12,626 | 1 | 28,593 | 3 | 27,820 | 1 |
|  | 17,905-19,772 |  | 13,045-17,588 |  | 7,259-12,638 |  | 9,435-16,250 |  | 25,874-31,589 |  | 23,612-31,981 |  |
| Bosnia | 16,240 | 1 | 19,631 | 9 | 11,497 | 4 | 14,636 | 7 | 27,737 | 1 | 34,267 | 9 |
|  | 16,051-16,428 |  | 18,676-20,644 |  | 8,598-14,754 |  | 11,014-18,694 |  | 24,837-31,016 |  | 30,469-38,499 |  |
| Bulgaria | 25,598 | 10 | 26,877 | 13 | 13,017 | 10 | 14,797 | 10 | 38,615 | 10 | 41,674 | 13 |
|  | 25,429-25,782 |  | 25,581-28,134 |  | 9,665-16,765 |  | 11,135-19,013 |  | 35,311-42,306 |  | 37,764-45,936 |  |
| Croatia | 22,957 | 6 | 18,941 | 8 | 12,696 | 8 | 14,762 | 9 | 35,652 | 6 | 33,702 | 8 |
|  | 22,749-23,174 |  | 18,017-19,942 |  | 9,520-16,294 |  | 11,114-18,915 |  | 32,487-39,284 |  | 30,101-37,886 |  |
| Czech Republic | 25,756 | 11 | 17,081 | 3 | 13,131 | 12 | 15,043 | 11 | 38,887 | 11 | 32,124 | 5 |
|  | 25,594-25,917 |  | 16,248-17,975 |  | 9,812-16,965 |  | 11,310-19,398 |  | 35,663-42,622 |  | 28,335-36,645 |  |
| Hungary | 31,030 | 13 | 21,949 | 10 | 14,115 | 13 | 15,106 | 12 | 45,145 | 13 | 37,055 | 11 |
|  | 30,856-31,207 |  | 20,963-22,982 |  | 10,658-18,134 |  | 11,405-19,390 |  | 41,647-49,139 |  | 33,211-41,267 |  |
| N. Macedonia | 20,049 | 5 | 17,518 | 4 | 11,101 | 2 | 13,312 | 2 | 31,150 | 4 | 30,829 | 3 |
|  | 19,703-20,383 |  | 16,684-18,389 |  | 8,293-14,288 |  | 9,945-17,124 |  | 28,357-34,339 |  | 27,400-34,655 |  |
| Montenegro | 16,677 | 2 | 18,803 | 7 | 11,132 | 3 | 13,363 | 3 | 27,809 | 2 | 32,166 | 6 |
|  | 16,272-17,110 |  | 17,518-20,166 |  | 8,348-14,408 |  | 9,988-17,154 |  | 24,951-31,076 |  | 28,728-36,262 |  |
| **Poland** | **23,423** | **7** | **18,474** | **6** | **12,238** | **6** | **14,268** | **6** | **35,661** | **7** | **32,742** | **7** |
|  | **23,346-23,505** |  | **17,665-19,325** |  | **9,156-15,837** |  | **10,667-18,310** |  | **32,576-39,220** |  | **29,058-36,867** |  |
| Romania | 26,267 | 12 | 24,249 | 12 | 13,069 | 11 | 14,647 | 8 | 39,336 | 12 | 38,896 | 12 |
|  | 26,144-26,386 |  | 23,199-25,256 |  | 9,788-16,776 |  | 11,040-18,870 |  | 36,009-43,028 |  | 35,131-43,112 |  |
| Serbia | 24,970 | 9 | 22,823 | 11 | 12,489 | 7 | 14,149 | 5 | 37,459 | 9 | 36,971 | 10 |
|  | 24,772-25,162 |  | 21,779-23,817 |  | 9,373-16,093 |  | 10,654-18,167 |  | 34,339-41,100 |  | 33,398-41,195 |  |
| Slovakia | 23,821 | 8 | 17,941 | 5 | 12,182 | 5 | 14,069 | 4 | 36,003 | 8 | 32,010 | 4 |
|  | 23,626-24,026 |  | 17,081-18,844 |  | 9,120-15,690 |  | 10,557-18,125 |  | 32,916-39,508 |  | 28,386-36,056 |  |
| Slovenia | 19,837 | 4 | 14,794 | 1 | 12,918 | 9 | 15,313 | 13 | 32,755 | 5 | 30,107 | 2 |
|  | 19,569-20,083 |  | 13,947-15,697 |  | 9,634-16,563 |  | 11,514-19,655 |  | 29,462-36,457 |  | 26,357-34,517 |  |

| **Males** | | | | | | | | | | | | |
| --- | --- | --- | --- | --- | --- | --- | --- | --- | --- | --- | --- | --- |
| **Central Europe** | **30,401** |  | **24,861** |  | **12,199** |  | **14,092** |  | **42,600** |  | **38,953** |  |
|  | **30,324-30,475** |  | **24,243-25,475** |  | **9,150-15,679** |  | **10,570-18,051** |  | **39,506-46,066** |  | **35,367-42,983** |  |
| Albania | 21,773 | 3 | 19,630 | 2 | 9,585 | 1 | 12,466 | 1 | 31,357 | 3 | 32,096 | 1 |
|  | 20,757-22,833 |  | 15,961-23,785 |  | 7,108-12,337 |  | 9,277-16,020 |  | 28,735-34,245 |  | 26,949-37,753 |  |
| Bosnia | 19,560 | 1 | 22,737 | 7 | 11,050 | 4 | 14,529 | 11 | 30,609 | 1 | 37,266 | 8 |
|  | 19,301-19,817 |  | 21,267-24,378 |  | 8,269-14,144 |  | 10,954-18,532 |  | 27,886-33,738 |  | 33,434-41,498 |  |
| Bulgaria | 32,358 | 12 | 32,930 | 13 | 12,894 | 12 | 14,244 | 8 | 45,252 | 12 | 47,174 | 13 |
|  | 32,099-32,629 |  | 30,821-35,008 |  | 9,640-16,565 |  | 10,722-18,302 |  | 41,930-48,881 |  | 43,127-51,706 |  |
| Croatia | 28,192 | 6 | 22,742 | 8 | 12,211 | 7 | 14,402 | 10 | 40,403 | 6 | 37,143 | 7 |
|  | 27,899-28,492 |  | 21,260-24,210 |  | 9,131-15,706 |  | 10,817-18,468 |  | 37,312-43,925 |  | 33,569-41,300 |  |
| Czech Republic | 31,538 | 11 | 20,312 | 3 | 12,396 | 9 | 14,764 | 12 | 43,934 | 10 | 35,076 | 5 |
|  | 31,320-31,765 |  | 19,010-21,738 |  | 9,305-16,028 |  | 11,081-18,987 |  | 40,784-47,576 |  | 31,229-39,432 |  |
| Hungary | 38,855 | 13 | 26,589 | 11 | 13,374 | 13 | 14,360 | 9 | 52,229 | 13 | 40,948 | 11 |
|  | 38,599-39,111 |  | 25,007-28,392 |  | 10,080-17,169 |  | 10,856-18,371 |  | 48,954-56,025 |  | 37,044-45,311 |  |
| N. Macedonia | 23,178 | 4 | 21,678 | 4 | 10,977 | 3 | 13,169 | 3 | 34,155 | 4 | 34,847 | 3 |
|  | 22,750-23,602 |  | 20,264-23,123 |  | 8,165-14,097 |  | 9,825-16,932 |  | 31,413-37,374 |  | 31,186-38,766 |  |
| Montenegro | 20,229 | 2 | 21,946 | 6 | 10,797 | 2 | 13,025 | 2 | 31,026 | 2 | 34,971 | 4 |
|  | 19,664-20,807 |  | 19,811-24,351 |  | 8,089-13,936 |  | 9,725-16,680 |  | 28,218-34,152 |  | 30,970-39,324 |  |
| **Poland** | **29,739** | **7** | **23,480** | **9** | **11,788** | **5** | **13,979** | **6** | **41,528** | **7** | **37,459** | **9** |
|  | **29,626-29,853** |  | **22,064-24,972** |  | **8,815-15,176** |  | **10,458-17,945** |  | **38,586-44,949** |  | **33,549-41,659** |  |
| Romania | 31,562 | 10 | 29,911 | 12 | 12,797 | 11 | 14,143 | 7 | 44,359 | 11 | 44,054 | 12 |
|  | 31,395-31,739 |  | 28,185-31,755 |  | 9,615-16,437 |  | 10,601-18,140 |  | 41,148-47,983 |  | 39,940-48,473 |  |
| Serbia | 31,031 | 9 | 25,007 | 10 | 12,219 | 8 | 13,729 | 4 | 43,250 | 9 | 38,736 | 10 |
|  | 30,784-31,286 |  | 23,486-26,553 |  | 9,160-15,668 |  | 10,349-17,560 |  | 40,206-46,673 |  | 35,036-42,918 |  |
| Slovakia | 30,387 | 8 | 21,826 | 5 | 11,931 | 6 | 13,804 | 5 | 42,319 | 8 | 35,630 | 6 |
|  | 30,112-30,663 |  | 20,501-23,240 |  | 8,954-15,386 |  | 10,300-17,766 |  | 39,246-45,795 |  | 31,976-39,966 |  |
| Slovenia | 24,671 | 5 | 17,946 | 1 | 12,448 | 10 | 15,010 | 13 | 37,119 | 5 | 32,956 | 2 |
|  | 24,324-25,041 |  | 16,571-19,322 |  | 9,264-15,977 |  | 11,235-19,275 |  | 34,016-40,749 |  | 29,127-37,419 |  |
| **Females** | | | | | | | | | | | | |
| **Central Europe** | **18,900** |  | **16,037** |  | **13,008** |  | **14,837** |  | **31,908** |  | **30,873** |  |
|  | **18,838-18,965** |  | **15,597-16,470** |  | **9,717-16,796** |  | **11,116-19,120** |  | **28,608-35,683** |  | **27,186-35,038** |  |
| Albania | 15,714 | 4 | 10,715 | 1 | 9,947 | 1 | 12,788 | 1 | 25,661 | 3 | 23,503 | 1 |
|  | 14,874-16,576 |  | 8,384-13,328 |  | 7,409-12,931 |  | 9,586-16,512 |  | 22,906-28,661 |  | 19,469-27,842 |  |
| Bosnia | 12,923 | 1 | 16,637 | 9 | 11,945 | 4 | 14,739 | 7 | 24,868 | 2 | 31,376 | 9 |
|  | 12,707-13,159 |  | 15,465-17,917 |  | 8,932-15,342 |  | 11,051-18,875 |  | 21,850-28,310 |  | 27,525-35,507 |  |
| Bulgaria | 19,006 | 9 | 21,166 | 13 | 13,137 | 8 | 15,316 | 11 | 32,143 | 10 | 36,484 | 13 |
|  | 18,790-19,235 |  | 19,799-22,682 |  | 9,788-16,970 |  | 11,487-19,730 |  | 28,833-35,907 |  | 32,257-40,831 |  |
| Croatia | 18,011 | 8 | 15,365 | 7 | 13,154 | 9 | 15,101 | 8 | 31,164 | 8 | 30,466 | 8 |
|  | 17,710-18,319 |  | 14,202-16,587 |  | 9,858-16,854 |  | 11,414-19,332 |  | 27,812-34,970 |  | 26,659-34,829 |  |
| Czech Republic | 20,301 | 11 | 13,952 | 5 | 13,824 | 12 | 15,313 | 10 | 34,125 | 11 | 29,265 | 6 |
|  | 20,097-20,522 |  | 12,987-1,030 |  | 10,323-17,779 |  | 11,521-19,787 |  | 30,744-37,994 |  | 25,309-33,883 |  |
| Hungary | 23,797 | 13 | 17,740 | 10 | 14,800 | 13 | 15,782 | 13 | 38,597 | 13 | 33,522 | 10 |
|  | 23,590-24,002 |  | 16,570-19,039 |  | 11,100-19,050 |  | 11,903-20,338 |  | 34,924-42,812 |  | 29,412-37,904 |  |
| N. Macedonia | 16,886 | 5 | 13,293 | 3 | 11,225 | 2 | 13,457 | 2 | 28,111 | 4 | 26,749 | 2 |
|  | 16,499-17,265 |  | 12,443-14,127 |  | 8,362-14,468 |  | 10,084-17,378 |  | 25,271-31,343 |  | 23,160-30,657 |  |
| Montenegro | 13,154 | 2 | 15,726 | 8 | 11,465 | 3 | 13,695 | 3 | 24,619 | 1 | 29,420 | 7 |
|  | 12,751-13,612 |  | 14,441-17,086 |  | 8,575-14,876 |  | 10,260-17,649 |  | 21,650-28,011 |  | 25,527-33,496 |  |
| **Poland** | **17,410** | **6** | **13,780** | **4** | **12,666** | **6** | **14,539** | **5** | **30,076** | **7** | **28,318** | **4** |
|  | **17,315-17,517** |  | **12,918-14,740** |  | **9,434-16,402** |  | **10,870-18,646** |  | **26,843-33,744** |  | **24,512-32,379** |  |
| Romania | 21,120 | 12 | 18,871 | 11 | 13,335 | 10 | 15,126 | 9 | 34,455 | 12 | 33,997 | 11 |
|  | 20,960-21,274 |  | 17,639-20,054 |  | 9,957-17,229 |  | 11,395-19,506 |  | 31,082-38,278 |  | 30,054-38,444 |  |
| Serbia | 19,106 | 10 | 20,675 | 12 | 12,751 | 7 | 14,561 | 6 | 31,857 | 9 | 35,236 | 12 |
|  | 18,887-19,322 |  | 19,266-22,081 |  | 9,542-16,454 |  | 10,999-18,701 |  | 28,696-35,550 |  | 31,536-39,299 |  |
| Slovakia | 17,547 | 7 | 14,240 | 6 | 12,422 | 5 | 14,321 | 4 | 29,970 | 6 | 28,560 | 5 |
|  | 17,299-17,780 |  | 13,202-15,346 |  | 9,271-16,076 |  | 10,768-18,459 |  | 26,835-33,664 |  | 24,960-32,619 |  |
| Slovenia | 15,259 | 3 | 11,687 | 2 | 13,364 | 11 | 15,613 | 12 | 28,623 | 5 | 27,300 | 3 |
|  | 14,900-15,626 |  | 10,663-12,789 |  | 9,992-17,230 |  | 11,724-20,044 |  | 25,253-32,452 |  | 23,373-31,765 |  |
