# Supplementary material for: Changes in disease burden in Poland between 1990–2017 in comparison with other Central European countries: A systematic analysis for the Global Burden of Disease Study 2017
Source: PLoS One. 2020 Mar 2;15(3):e0226766. doi: 10.1371/journal.pone.0226766 (PMC7051048; doi:10.1371/journal.pone.0226766)
Supplement: S2 Table — (DOCX) [file pone.0226766.s002.docx]

S2 Table. All-age rates, percent contribution, and relative (%) change for Level 2 causes of YLLs, YLDs and DALYs for Poland and Central Europe, both sexes combined, in 1990 and 2017.

| **All-age YLL rate** | | | | | | **All-age YLD rate** | | | | | |  | **All-age DALY rate** | | | | |
| --- | --- | --- | --- | --- | --- | --- | --- | --- | --- | --- | --- | --- | --- | --- | --- | --- | --- |
| **Cause** | Change in rank | 1990 | | 2017 | | **Cause** | Change in rank | 1990 | | 2017 | | **Cause** | Change in rank | 1990 | | 2017 | |
|  |  | Rate per 100,000 | Contribution % (95% UI) | Rate per 100,000 | Change % |  |  | Rate per 100,000 | Contribution % (95% UI) | Rate per 100,000 | Change % |  |  | Rate per 100,000 | Contribution % (95% UI) | Rate per 100,000 | Change % |
|  |  |  | | | |  |  | Poland | | | |  |  |  | | | |
| Cardiovascular diseases | 0 | 9,402.08 | 33.8  33.2-34.5 | 6,249.23 | -33.5 | Musculoskeletal disorders | 0 | 1,888.59 | 15.4 13.5-17.5 | 2,452.07 | 17.2 | Cardiovascular diseases | 0 | 10,123.85 | 22.1  20.2-24.1 | 7,231.77 | -28.6 |
| Neoplasms | 0 | 5,018.85 | 32.2  31.5-32.8 | 5,948.05 | 18.5 | Unintentional injuries | 0 | 1,630.58 | 13.3 12.0-17.8 | 1,908.50 | 13.3 | Neoplasms | 0 | 5,112.9 | 18.8  16.8-20.9 | 6,125.29 | 19.8 |
| Digestive diseases | +4 | 818.65 | 5.5  5.3-5.8 | 1,020.41 | 24.6 | Mental disorders | 0 | 1,324.43 | 10.8 9.2-12.3 | 1,420.18 | 10.0 | Unintentional injuries | 0 | 2,701.68 | 7.8  6.7-9.0 | 2,553.22 | -5.5 |
| Neurological disorders | +8 | 601.63 | 4.9  4.8-5.1 | 905.73 | 50.6 | Neurological disorders | 0 | 1,025.53 | 8.4 6.3-10.7 | 1,197.31 | 8.4 | Musculoskeletal disorders | 0 | 1,928.7 | 7.6  6.1-9.1 | 2,482.12 | 28.7 |
| Self-harm & violence | +3 | 793.02 | 4.2  4.0-4.4 | 772.31 | -2.6 | Sense organ diseases | +1 | 839.14 | 6.8 5.5-8.5 | 1,106.04 | 7.7 | Neurological disorders | +1 | 1,627.15 | 6.4  5.6-7.4 | 2,103.04 | 29.3 |
| Unintentional injuries | -1 | 1,071.1 | 3.5  3.4-.3.6 | 644.72 | -39.8 | Cardiovascular diseases | +1 | 721.78 | 5.9 5.2-6.6 | 982.54 | 6.9 | Digestive diseases | +5 | 1,267.74 | 4.7  4.4-5.0 | 1,531.24 | 20.8 |
| Respiratory infections &TB | +2 | 713.74 | 2.9  2.7-3.0 | 531.19 | -25.6 | Other non-communicable | -2 | 848.26 | 6.9  5.8-8.3 | 894.73 | 6.3 | Mental disorders | +3 | 1,324.71 | 4.3  3.5-5.2 | 1,420.81 | 7.3 |
| Chronic respiratory | +2 | 676.96 | 2.6  2.5-2.8 | 485.57 | -28.3 | Diabetes & CKD | +1 | 503.25 | 4.1 3.6-4.7 | 805.95 | 5.6 | Diabetes & CKD | +4 | 1,111.84 | 3.8  3.4-4.3 | 1,261.08 | 13.4 |
| Transport injuries | -6 | 1,214.24 | 2.5  2.4-2.6 | 464.08 | -61.8 | Chronic respiratory | -1 | 681.76 | 5.6  4.7-6.7 | 729.58 | 5.2 | Chronic respiratory | 0 | 1,358.72 | 3.7  3.4-4.1 | 1,215.15 | -10.6 |
| Diabetes & CKD | +1 | 608.58 | 2.5  2.4-2.6 | 455.12 | -25.2 | Skin  diseases | +1 | 497.29 | 4.1  3.2-5.1 | 512.45 | 3.6 | Other non-communicable | -5 | 1,723.77 | 3.5  2.9-4.3 | 1,162.31 | -32.6 |
| Substance use | +2 | 297.39 | 2.5  2.3-2.7 | 452.76 | 52.2 | Digestive diseases | +1 | 449.09 | 3.6  3.1-4.2 | 510.83 | 3.6 | Sense organ diseases | +3 | 839.14 | 3.4  2.5-4.4 | 1,106.04 | 31.8 |
| Other non-communicable | -6 | 875.5 | 1.5  1.3-1.7 | 267.58 | -69.4 | Maternal & neonatal | -2 | 499.34 | 4.1 2.8-5.7 | 486.23 | 3.5 | Self-harm & violence | +1 | 849.02 | 2.6  2.3-2.8 | 834.52 | -1.7 |
| Maternal & neonatal | -9 | 1,080.26 | 0.9  0.7-1.1 | 162.26 | -85.0 | Substance use | +1 | 307.00 | 2.5  2.1-3.0 | 365.49 | 2.6 | Substance use | +3 | 604.39 | 2.5  2.3-2.7 | 818.25 | 35.4 |
| Other infections | 0 | 154.58 | 0,2  0,2-0,2 | 36.44 | -76.4 | Transport injuries | -1 | 323.65 | 2.6  2.4-2.9 | 332.38 | 2.3 | Transport injuries | -6 | 1,537.89 | 2.4  2.3-2.6 | 796.46 | -48.2 |
| Musculoskeletal disorders | 0 | 40.11 | 0.2  0.2-0.2 | 30.06 | -25.1 | Neoplasms | +2 | 93.35 | 0.8  0.7-0.8 | 177.23 | 1.2 | Maternal & neonatal | -8 | 1579.61 | 2.0  1.5-2.6 | 648.49 | -59.0 |
| HIV/AIDS & STIs | +1 | 14.36 | 0.1  0.1-0.1 | 18.23 | 27.0 | Nutritional deficiencies | -1 | 299.36 | 2.4  1.7-3.3 | 101.19 | 0.7 | Respiratory infections &TB | -1 | 817.07 | 1.9  1.7-2.1 | 620.46 | -24.1 |
| Enteric infections | -1 | 26.97 | 0.1  0.1-0.1 | 12.87 | -52.3 | Respiratory infections &TB | -1 | 103.33 | 0.8  0.6-1.1 | 89.27 | 0.6 | Skin  diseases | 0 | 505.45 | 1.6  1.2-2.2 | 522.87 | 3.5 |
| Skin  diseases | 0 | 8.16 | 0.1  0.0-0.1 | 10.41 | 27.6 | Enteric infections | 0 | 70.45 | 0.6  0.5-0.7 | 64.00 | 0.5 | Nutritional deficiencies | 0 | 303.43 | 0.3  0.2-0.5 | 106.84 | -64.8 |
| Nutritional deficiencies | 0 | 4.08 | 0.0  0.0-0.0 | 5.65 | 38.6 | Self-harm & violence | 0 | 56.00 | 0.5  0.4-0.5 | 62.22 | 0.4 | Enteric infections | +1 | 97.42 | 0.2  0.2-0.3 | 76.86 | -21.1 |
| Mental disorders | +1 | 0.27 | 0.0  0.0-0.0 | 0.63 | 130.4 | NTDs & malaria | 0 | 30.30 | 0.3  0.1-0.4 | 31.90 | 0.2 | Other infections | -1 | 182.04 | 0.2  0.2-0.2 | 53.7 | -70.5 |
| NTDs & malaria | -1 | 2.49 | 0.0  0.0-0.0 | 0.56 | -77.4 | HIV/AIDS & STIs | +1 | 18.10 | 0.2  0.1-0.3 | 20.35 | 0.1 | HIV/AIDS & STIs | +1 | 32.46 | 0.1  0.1-0.2 | 38.58 | 18.9 |
|  |  |  |  |  |  | Other infections | -1 | 27.46 | 0.2  0.2-0.3 | 17.26 | 0.1 | NTDs & malaria | -1 | 32.79 | 0.1  0.0-0.2 | 32.47 | -1.0 |

|  |  | Central Europe | | | | | | | | | | | | | | | |
| --- | --- | --- | --- | --- | --- | --- | --- | --- | --- | --- | --- | --- | --- | --- | --- | --- | --- |
| Cardiovascular diseases | 0 | 9,892.75 | 39.9  39.5-40.3 | 8,106.1 | -18.1 | Musculoskeletal disorders | 0 | 2,007.65 | 15.9  14.0—18.0 | 2,511.36 | 17.3 | Cardiovascular diseases | 0 | 10,707.93 | 26.5  24.2-28.6 | 9,192.04 | -14.2 |
| Neoplasms | 0 | 4,960.74 | 29.0  28.5-29.3 | 5,898.49 | 18.9 | Unintentional injuries | 0 | 1,680,29 | 13.3  12.0-14.9 | 1,876.86 | 12.9 | Neoplasms | 0 | 5,067.22 | 17.6  15.8-19.4 | 6,090.6 | 20.2 |
| Digestive diseases | 0 | 1,189.18 | 5.7  4.5-5.8 | 1,150.79 | -3.2 | Mental disorders | 0 | 1,415.28 | 11.2  9.5-12.8 | 1,471.44 | 10.2 | Musculoskeletal disorders | +1 | 2,032.81 | 7.3  5.9-8.8 | 2,531.2 | 24.8 |
| Neurological disorders | +7 | 599.53 | 4.4  4.3-4.5 | 902.73 | 50.6 | Neurological disorders | 0 | 1,045.40 | 8.3  6.4-10.6 | 1,191.23 | 8.2 | Unintentional injuries | -1 | 2,859.34 | 7.1  6.1-8.3 | 2,492.61 | -12.8 |
| Self-harm & violence | +3 | 876.29 | 3.1  3.0-3.2 | 637.44 | -27.3 | Sense organ diseases | 0 | 853.00 | 6.8  5.4-8.5 | 1,122.43 | 7.7 | Neurological disorders | +1 | 1,644.93 | 6.0  5.3-6.9 | 2,093.96 | 27.3 |
| Unintentional injuries | -2 | 1,179.06 | 3.0  3.0-3.1 | 615.75 | -47.8 | Cardiovascular diseases | +1 | 815.17 | 6.5  5.7-7.3 | 1,085.94 | 7.5 | Digestive diseases | +1 | 1,617.82 | 4.7  4.4-4.9 | 1,619.52 | 0.1 |
| Diabetes & CKD | +5 | 579.89 | 3.0  2.9-3.0 | 606.51 | 4.6 | Diabetes & CKD | +2 | 563.12 | 4.5  4.0-5.0 | 886.93 | 6.1 | Diabetes & CKD | +6 | 1,143.01 | 4.3  3.9-4.8 | 1,493.45 | 30.7 |
| Chronic respiratory | +2 | 725.46 | 2.9  2.9-3.0 | 588.81 | -18.8 | Other non-communicable | -2 | 828.01 | 6.6  5.5-7.8 | 876.70 | 6.0 | Mental disorders | +1 | 1,415.42 | 4.2  3.4-5.0 | 1,471.74 | 4.0 |
| Respiratory infections &TB | -4 | 1,106.13 | 2.6  2.6-2.7 | 535.11 | -51.6 | Chronic respiratory | -1 | 654.50 | 5.2  4.4-6.3 | 722.20 | 5.0 | Chronic respiratory | +1 | 1,379.96 | 3.8  3.4-4.1 | 1,311 | -5.0 |
| Transport injuries | -4 | 1,005.7 | 2.1  2.0-2.2 | 424.69 | -57.8 | Skin  diseases | 0 | 479.85 | 3.8  3.0-4.8 | 493.82 | 3.4 | Other non-communicable | -5 | 1,681.52 | 3.3  2.7-3.9 | 1,144.26 | -32.0 |
| Other non-communicable | -2 | 853.51 | 1.3  1.2-1.4 | 267.55 | -68.7 | Digestive diseases | +1 | 428.64 | 3.4  2.9-3.9 | 468.73 | 3.2 | Sense organ diseases | +4 | 853 | 3.2  2.4-4.2 | 1,122.43 | 31.6 |
| Substance use | +1 | 232.28 | 1.3  1.2-1.4 | 262.21 | 12.9 | Maternal & neonatal | -1 | 442.69 | 3.6  2.5-4.8 | 433.06 | 3.0 | Transport injuries | -1 | 1,306.64 | 2.1  2.0-2.2 | 737.84 | -43.5 |
| Maternal & neonatal | -6 | 995.53 | 1.0  0.9-1.1 | 196.64 | -80.3 | Substance use | +1 | 311.78 | 2.5  2.0-2.9 | 341.11 | 2.4 | Self-harm & violence | +1 | 942.26 | 2.1  1.9-2.3 | 718.1 | -23.8 |
| Other infections | 0 | 203.62 | 0.25  0.2-0.3 | 51.6 | -74.7 | Transport injuries | +1 | 300.94 | 2.4  2.2-2.6 | 313.14 | 2.2 | Maternal & neonatal | -6 | 1,438.22 | 1.8  1.5-2.2 | 629.69 | -56.2 |
| Musculoskeletal disorders | +2 | 25.16 | 0.1  0.1-0.1 | 25.84 | 2.71 | Neoplasms | +1 | 106.47 | 0.8  0.8-0.9 | 192.11 | 1.3 | Respiratory infections &TB | -3 | 1,209.76 | 1.8  1.7-2.0 | 626.2 | -48.2 |
| HIV/AIDS & STIs | 0 | 31.11 | 0.1  0.1-0.1 | 23.35 | -25.0 | Nutritional deficiencies | -3 | 340.39 | 2.7  2.0-3.5 | 161.12 | 1.1 | Substance use | 0 | 544.06 | 1.7  1.6-1.9 | 603.32 | 10.9 |
| Enteric infections | -2 | 56.79 | 0.1  0.1-0.1 | 19.91 | -65.0 | Respiratory infections &TB | 0 | 103.63 | 0.8  0.6-1.1 | 91.10 | 0.6 | Skin  diseases | 0 | 489.16 | 1.5  1.1-2.0 | 506.3 | 3.5 |
| Skin  diseases | 0 | 9.31 | 0.1  0.0-0.1 | 12.47 | 34.0 | Enteric infections | 0 | 86.76 | 0.7  0.6-0.8 | 80.94 | 0.6 | Nutritional deficiencies | 0 | 347.57 | 0.5  0.3-0.7 | 168.26 | -51.6 |
| Nutritional deficiencies | 0 | 7.17 | 0.0  0.0-0.0 | 7.14 | -0.4 | Self-harm & violence | 0 | 65.97 | 0.5  0.5-0.6 | 80.65 | 0.6 | Enteric infections | +1 | 143.55 | 0.3  0.2-0.3 | 100.85 | -29.8 |
| NTDs & malaria | 0 | 6.13 | 0.0  0.0-0.0 | 1.14 | -81.5 | NTDs & malaria | 0 | 31.76 | 0.3  0.2-0.4 | 30.54 | 0.2 | Other infections | -1 | 235.11 | 0.2  0.2-0.2 | 73.47 | -68.8 |
| Mental disorders | 0 | 0.14 | 0.0  0.0-0.0 | 0.31 | 16.1 | Other infections | 0 | 31.49 | 0.3  0.2-0.3 | 21.87 | 0.2 | HIV/AIDS & STIs | 0 | 49.76 | 0.1  0.1-0.2 | 43.99 | -11.6 |
|  |  |  |  |  |  | HIV/AIDS & STIs | 0 | 18.65 | 0.2  0.1-0.3 | 20.63 | 0.1 | NTDs & malaria | 0 | 37.89 | 0.1  0.1-0.1 | 31.67 | -16.4 |

. Colors indicate different groups of causes: red = CMMN, blue = NCD, green = Injuries
