# Supplementary material for: Changes in disease burden in Poland between 1990–2017 in comparison with other Central European countries: A systematic analysis for the Global Burden of Disease Study 2017
Source: PLoS One. 2020 Mar 2;15(3):e0226766. doi: 10.1371/journal.pone.0226766 (PMC7051048; doi:10.1371/journal.pone.0226766)
Supplement: S1 Fig — Top 25 Level 3 causes of YLLs (a), YLDs (b), and DALYs (c) in the CE region for both sexes combined in 1990 and 2017: (a) YLLs, (b) YLDs, (c) DALYs. Conditions are ranked according to age-standardized rates, from highest to lowest. Colors indicate changes in rank: red = increase, green = decrease, and purple = no change. The numbers are percentage changes in counts, all-age rates, and age-standardized rates. (DOCX) [file pone.0226766.s003.docx]

S1 Fig. Top 25 Level 3 causes of YLLs (a), YLDs (b), and DALYs (c) in the CE region for both sexes combined in 1990 and 2017

1. YLLs


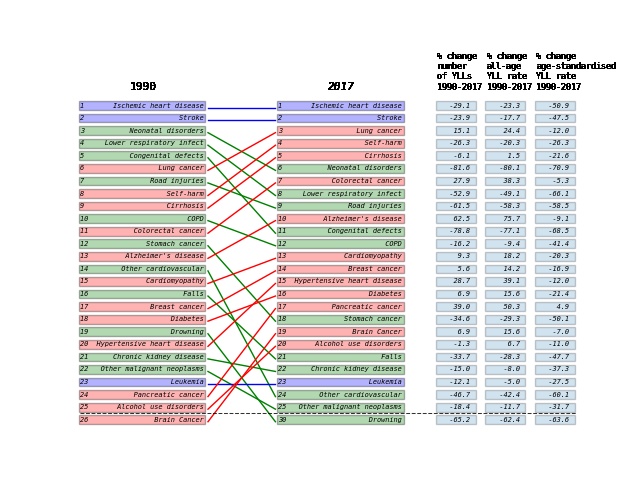


1. YLDs


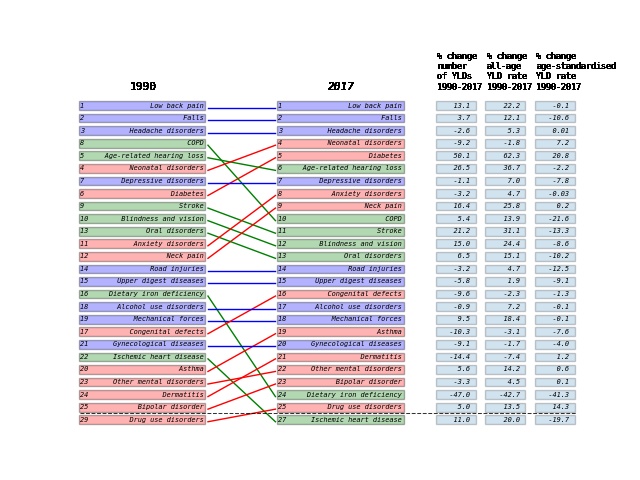


1. DALYs


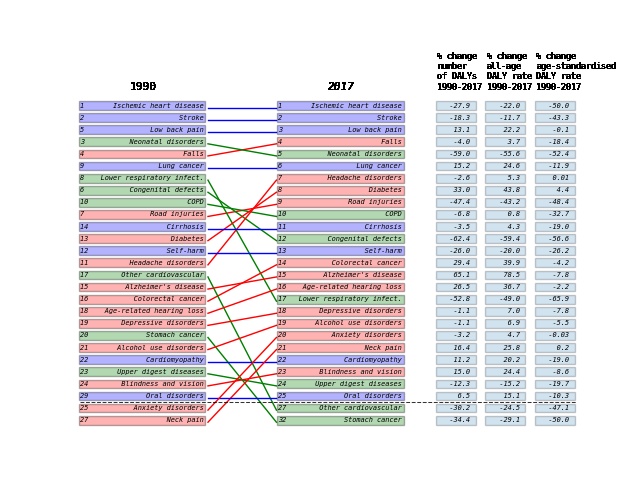


Conditions are ranked according to age-standardized rates, from highest to lowest. Colors indicate changes in rank: red = increase, green = decrease, and purple = no change. The numbers are percentage changes in counts, all-age rates, and age-standardized rates.
